# Health inequities faced by sex workers

**DOI:** 10.1016/S2468-2667(25)00307-X

**Published:** 2026-07-24

**Authors:** Katherine Rucinski, Mary Anne Roach, Anna Shapiro, Yigit Aydinalp, Kaitlyn Atkins, Joseph G Rosen, Karina Bravo Neira, Niki Lovisa, Trajche Janushev, Stefan Baral, Shira Goldenberg

**Affiliations:** Johns Hopkins Bloomberg School of Public Health, Baltimore, MD, USA; Johns Hopkins Bloomberg School of Public Health, Baltimore, MD, USA; Global Network of Sex Work Projects, Edinburgh, UK; University of Sheffield, Sheffield, UK; Global Network of Sex Work Projects, Edinburgh, UK; Johns Hopkins Bloomberg School of Public Health, Baltimore, MD, USA; Division of General Internal Medicine, Rhode Island Hospital, Providence, RI, USA; Department of Medicine, Warren Alpert Medical School, Brown University, Providence, RI, USA; Department of Epidemiology, School of Public Health, Brown University, Providence, RI, USA; Plataforma Latinoamericana de Personas que Ejercen el Trabajo Sexual, Machala, Ecuador; Guyana Vulnerable Population Alliance, Georgetown, Guyana; Sex Workers’ Rights Advocacy Network, Budapest, Hungary; Johns Hopkins Bloomberg School of Public Health, Baltimore, MD, USA; School of Public Health, San Diego State University, San Diego, CA, USA

## Abstract

Stark health inequities persist for sex workers globally—particularly in the areas of mental health, sexual and reproductive health, and harm reduction. To inform this Series paper we reviewed epidemiological studies published over the past decade to describe the burden of key health outcomes among sex workers and associated factors contributing to these disparities. Within the mental health literature, we estimate at least one in four and as many as four in five sex workers experience depression. Within sexual and reproductive health literature, at least one in three sex workers are estimated to experience an unmet need for contraception. Using a Health Equity framework, we contextualise evidence gaps related to these health areas across multiple levels of influence (eg, systems of power, relationships and networks, physiological pathways, and individual factors) and offer recommendations to advance an agenda of health equity for sex workers in future research and programmes. Priority programme and policies include upholding the autonomy and dignity of sex worker communities, ensuring the inclusion of gender diverse populations in research, addressing structural determinants of health, and fostering meaningful community engagement and collaboration.

## Introduction

A substantial body of evidence has shown that sex workers (people aged 18 years or older of any gender who receive money, goods, or reward in exchange for consensual sexual services) either regularly or occasionally globally face disproportionate inequities in HIV and other sexually transmitted infections (STIs),^1,2^ as well as violence and other human rights violations.^3^ Much of this research has focused explicitly on sexual health, with outcomes largely centred on the prevention and treatment cascades for HIV and other STIs, including incidence, prevalence, testing, and suboptimal access to and outcomes of antiretroviral therapy.^4,5^ However, sex workers represent a population with far broader health needs that have received comparably less attention—for example, few previous syntheses have attended to other areas of mental health, sexual and reproductive health (SRH) and rights, and harm reduction beyond the implications for HIV and other STIs.^6^

The health inequities faced by sex workers are deeply rooted in interconnected social and structural factors such as the explicit criminalisation of sex work (comprising either the buying or selling of sex, or both), stigma and discrimination, social injustices, and policies and practices that foster violations of sex workers’ health and human rights. For example, a systematic review and meta-analysis documented that in settings where sex work was criminalised, women had 40% increased likelihood of reporting increased HIV and STI risk exposures, such as condomless sex, as well as twice the likelihood of experiencing physical or sexual violence compared with sex workers in settings with less repressive policing and criminalisation practices.^7^ Other reviews have reported a disproportionately high burden of self-managed (thus higher-risk) abortion among sex workers, especially in more restrictive legal environments.^8^ Additional literature has focused on the burden of comorbid mental health conditions—namely depression, anxiety, post-traumatic stress disorder (PTSD), and suicidal ideation—among sex workers globally.^9^ Further, consistent evidence shows that stigma and discrimination, often acting synergistically with criminalisation and other repressive laws, policies, and practices, restrict sex workers’ access to life-saving health services and exacerbate health inequities.^10,11^ However, across these reviews, there has been relatively little exploration of how these widespread inequities challenge broader achievement of health equity among sex workers.

Although health services for sex workers have proliferated over the past decade, services are often hyper-localised and not delivered at scale; insufficiently funded, coordinated, or supported by the public sector; inattentive to sex workers’ health needs beyond HIV and STIs and their determinants; and predominantly serve cisgender women.^12,8^ Addressing the health needs of sex workers of all genders requires a more holistic, equity-oriented approach that seeks to understand and address sex workers’ wellbeing in all their diversity as well as correcting the inherent injustice of poor health outcomes among sex workers, rather than simply treating or preventing health conditions.^8^ Further, improving health equity for sex workers necessitates sex-worker-led research alongside meaningful engagement with sex-worker-led organisations and sex worker communities.^13^ Sex workers are uniquely positioned to develop and guide research inquiries into their experiences.^14^

Improving health for sex workers requires a comprehensive understanding of inequities faced by sex workers globally. This Series paper is informed by a systematic review of epidemiological literature from the past decade (2015–25). Our synthesis aims to evaluate health equity among sex worker communities beyond HIV and STIs, with attention to outcomes and accessibility and uptake of services related to mental health, SRH, and harm reduction. Methods are detailed in the appendix (p 1).

We define sex work, as per sex worker rights advocates, as an occupation with provision of services in exchange for a negotiated price between consenting adults, distinct from casual transactional sex or sexual exploitation.^15^ In conducting this synthesis, we draw on the Health Equity Framework in considering the presence and temporal evolution (ie, life-course perspective) of structural, relational, individual, and physiological spheres and their influence on health outcomes, both between population strata and within sex worker populations.^16^ This framework was chosen for its conceptualisation of health equity in terms of not only individual agency but also fair access to (and allocation of) resources and opportunities to achieve health, which are particularly salient for sex worker communities given pervasive stigma and discrimination.^16^ Health equity resources are shaped by multilevel and interacting spheres of influence—spanning relationships and networks, systems of power, physiological pathways, and individual factors (figure). Individual factors include sex workers’ individual attributes and behaviours, such as resilience, self-efficacy, and personal health-seeking behaviours that affect health outcomes, however, these factors interact with other spheres. For example, systems of power include legal and policy factors, such as criminalisation of sex work, which can hinder access to justice and serve as barriers to health care, or resource allocations focused explicitly on HIV that fail to address sex workers’ health holistically. A summary of the peer-reviewed evidence is provided in the appendix (p 7).

Throughout this Series paper, the panels detail and contextualise the lived experiences of sex workers, in their own words.

## Mental health

Over the past decade, research has documented an elevated burden of mental health symptoms among sex workers globally, with depression being the most commonly assessed among studies in this synthesis.^7–20^ Reported prevalence of depressive symptoms among sex workers range from 8%^21,22^ to 90%,^23^ with most studies reporting prevalence exceeding one in four sex workers, with substantial heterogeneity by context and measurement instrument (appendix p 13). Prevalences of generalised anxiety and PTSD are also elevated among sex workers, although less studied than depressive symptoms. Studies of anxiety among sex workers report prevalence estimates ranging from 8%^24^ to 80%.^25^ Studies of PTSD among sex workers report prevalences ranging from 8%^21^ to 75%.^26^ Across outcomes, geographies, and populations, violence (including intimate partner violence, physical assault, sexual assault, and threats of violence) and stigma (including internalised, perceived, and enacted stigmas) are the most common risk factors for mental health symptoms among sex workers. For example, a 2017 cross-sectional study including 222 cisgender female sex workers in Mongolia^27^ found that 134 (60%) of participants had depressive symptoms, which increased linearly with exposure to sex work-related stigma (β=0·12, p=0·05). Comorbid symptoms of depression, anxiety, and PTSD have also been shown to be common in sex work communities. A 2022 cohort study with street-based cisgender and transgender female sex workers in England^28^ found recent violence from clients was associated with symptoms of depression and anxiety. A 2021 community-based cross-sectional study in South Africa^29^ similarly found that intimate partner violence was associated with increased depression and PTSD. Structural factors, including criminalisation and arrest, access to and control over economic resources, and housing and the built environment also contribute to mental health morbidity among sex workers.^30–32^ For example, a 2020 study with cisgender female sex workers in Cambodia^33^ found that both food and housing insecurity were associated with psychological distress. A 2015 study of cisgender female sex workers in India^34^ found increased symptoms of depression among sex workers with a history of arrest and those who reported lower autonomy over financial and household decisions.

Much of the recent literature on mental health among sex workers has focused on mental health and adversity, with few studies reporting on protective factors. However, where protective factors are evaluated, social support and resilient coping consistently emerge as being associated with lower levels of mental distress and can have a role in care-seeking.^27,35^ For example, a cross-sectional study of 199 transgender sex workers in China^35^ found that women needing psychiatric support who had adaptive coping were over three times more likely to intend to seek help than those with maladaptive coping, and that social support was associated with a 6% increased likelihood of intention to seek help among women with depression, anxiety, or suicidal ideation. Studies suggest uptake of mental health services are also lower among sex workers despite a clear burden of mental health symptoms among sex work communities. For example, a 2019 study of cisgender female sex workers in Switzerland found that 45 (75%) of 60 participants had at least one mental health problem, 20 (33%) had used mental health care for at least 1 of the past 6 months, and that stigma was a barrier to care-seeking.^36^

Emerging literature shows a crucial need to address suicidal ideation and behaviour among sex workers. There is substantial heterogeneity in measurement of suicidal ideation, with many studies using single items to capture thoughts of suicide over a given period, although validated tools are in use including the Suicidal Ideation Scale^25^ and the Suicidal Ideation Questionnaire.^37^ Reported prevalences of suicidal ideation among sex workers in the literature range from 3%^21^ to 46%.^37^ Literature on suicide attempts is scarce.^37,38^ One study collaborated with sex worker communities across eight low-income and middle-income countries (Angola, Brazil, DR Congo, India, Indonesia, Kenya, Nigeria, and South Africa) to examine causes of cisgender female sex worker mortality and found that 288 (14%) of 2112 sex worker deaths were attributable to suicide, of which 242 (84%) were reported in sub-Saharan Africa and 178 (62%) were defined as maternal suicides, occurring due to pregnancy or soon after birth.^39^

The testimony presented in panel 1 highlights the experience of a 38-year-old sex worker living with HIV who experienced severe depressive symptoms after receiving an HIV diagnosis.

## Sexual and reproductive health

Existing evidence regarding the SRH inequities faced by sex workers highlights major gaps in access to contraception, with insufficient data available on broader SRH outcomes including childbearing plans, infertility, human papillomavirus (HPV), and gender-affirming care (appendix pp 14–27). The unmet need for contraception or family planning has been assessed for sex workers across both high-income and low-income settings. Studies have generally defined the unmet need for contraception among cisgender women who are not actively using non-barrier contraceptive methods and who desire to prevent or delay pregnancy.^40^ Within the literature, the prevalence of unmet need for contraception ranged from 25% among hotel-based cisgender female sex workers in Bangladesh^41^ to 64% among cisgender female sex workers living with HIV in Kenya,^42^ with most studies documenting a burden of at least 30%. Similarly, the use of non-barrier modern contraceptive methods, including long-acting reversible contraception, ranged from 15% among cisgender female sex workers in South Africa^43^ to 95% among cisgender female sex workers enrolled in a health services intervention in Mozambique.^44^ International guidelines for sex worker SRH services highlight the crucial need for family planning and contraceptive counselling in the context of broader services given inadequate existing service provisions, scattered and siloed services, and insufficient access to the provision of contraceptive resulting from insurance coverage limits or broader structural concerns that preclude sex workers from accessing contraception through public health systems.^8^ Yet few data are available regarding sex workers’ long-term childbearing and parenting intentions, desires, and priorities, which are needed to inform interventions that are holistic and respect sex workers’ reproductive rights and autonomy. Among the few studies that assess the fertility intentions of sex workers (20 publications), a high proportion report wanting to have children in the future, ranging from 15% of cisgender female sex workers living in Mali or Benin^45^ to 64% of cisgender female sex workers in Cameroon.^46^ Of the five studies that reported on HPV, prevalence was high, ranging from 26% among cisgender female sex workers in Accra, Ghana,^47^ to 87% of sex workers living in Benin or Mali.^48^ Despite studies reporting consistently high HPV prevalence, cervical cancer screening uptake varied considerably from 0% of cisgender female sex workers reporting history of cervical cancer screening in Mozambique,^44,49^ to 77% of cisgender female sex workers reporting cervical cancer screening in the Dominican Republic.^50^ Although gender-affirming care is also essential for meeting the SRH needs of sex workers and has been reinforced through community literature and case studies,^8,51^ no studies explicitly describe uptake or unmet needs related to these services such as counselling and referrals for hormone therapy or other transgender-specific SRH services. Moreover, there is a gap in literature focused on the sexual health of sex workers more broadly, as most of these data exist within the context of HIV prevention and treatment studies and focused on related outcomes such as prevention of vertical transmission.

Despite the burden of maternal mortality and morbidity, there remains an insufficient body of research focused on access to and experiences with perinatal and postnatal care for sex worker communities. Within the literature, only five identified studies reported on antenatal care access, including an HIV-prevention randomised control trial in Tanzania,^52^ and an integrated biobehavioural survey for HIV monitoring in Brazil.^53,54^ Research on sex workers’ maternal health has primarily focused on concerns related to vertical transmission, with relatively less attention placed on sex worker wants and desires as mothers.^8^ Within this literature, however, important insights into access barriers have emerged. For example, one such study in South Africa found that the average time to initiating antenatal care was 19 weeks, often delayed to the third trimester due to late pregnancy detection, fear of maltreatment in health-care settings, perceived lack of partner and familial support, and a scarcity of knowledge of when to obtain antenatal care.^55^ These data align with a 2023 scoping review of maternal health service use among cisgender female sex workers,^56^ which found, among an extremely minimal evidence base of patterns of antenatal care use globally, that barriers to uptake of antenatal care spanned myriad factors including provider mistreatment and facility requirements of a male partner’s attendance. The testimony presented in panel 2 highlights some of the challenges pregnant and parenting sex workers face, including a general scarcity of social and economic support, mental health challenges, the complexities of acquiring perinatal and postnatal medication, and the potential for lost income if not working while pregnant. Community literature has reinforced the importance of wraparound, one-stop models for sex workers as a pathway to increase access to SRH information, including contraception, pregnancy testing, antenatal care, and abortion services.^8^ Expanding these models to include integrated health-care services, such as perinatal and postnatal care, could provide an opportunity to dismantle existing barriers to care and potentially reduce sex worker maternal morbidity and mortality, among other health benefits.

The literature indicates a high prevalence of abortion among sex workers; however, there is insufficient research examining how the criminalisation of abortion affects access to appropriate services and impacts broader violations of reproductive rights. Published estimates of lifetime prevalence of elective abortion among sex workers over the past 10 years ranges from 11% of sex workers in Benin^57^ to 93% in Viet Nam,^58^ with most studies reporting this to be at least one third of sex workers. Heterogeneity in abortion rates across settings have also been reported among other women of reproductive age more broadly, varying between 19 per 1000 women in Australia and New Zealand to 61 per 1000 women in west Asia and north Africa.^59^ In countries where abortion is restricted or criminalised, rates generally mirror those reported in places where abortion is more accessible with the caveat that women must often seek unsafe methods of abortion that result in a higher burden of complications, including death.^59,60^ Only one study from Zambia assessed abortion within varying structural contexts, and found that increased experience of arrest or incarceration was associated with an increased likelihood of abortion.^61^ Access to safe abortion and post-abortion services is crucial for sex worker communities amid insufficient access to contraceptives, experiences of sexual violence, and challenges negotiating the use of condoms.^8^ Positioning abortion and post-abortion care as a crucial health service that complements other SRH services is essential for ensuring that sex workers’ right to choose if and when they want to become parents is respected and affirmed.

## Drug-related harm reduction service access and needs

The evidence base on inequities faced by sex workers accessing harm reduction services is scarce and highlights important gaps in understanding the needs of sex workers who would benefit from these services. Although estimates vary widely by setting and work environment, sex workers who use drugs comprise an important subpopulation whose harm reduction needs remain under-addressed in research and programming. A 2025 systematic review found 46 countries reporting prevalence data on drug use among sex workers, with a 35% pooled prevalence of lifetime use of criminalised drugs among sex workers and estimates ranging from 1% to 84% across various countries, regions, and settings.^62^

Little research over the past 10 years has assessed sex workers’ access to harm reduction services, and much of this evidence has emanated from only three established cohorts of primarily cisgender female sex workers who use or inject drugs, or both, in urban North American settings (Canada,^63^ Mexico,^64^ and the USA^65^). The available evidence indicates tremendous unmet need for harm reduction services among sex workers. Estimates of access to syringe services programmes are heterogeneous; one study^66^ of 255 cisgender female sex workers reporting past-year illicit drug use in urban Kazakhstan reported 27 (11%) participants accessing a syringe services programme in their lifetimes, whereas a US study^67^ that analysed data from 175 cisgender female sex workers who injected drugs estimated that 113 (65%) accessed syringes from a syringe services programme, which was protective against receptive syringe sharing. Studies from Mexico have reported between 12% and 55% lifetime access to methadone treatment services in Cuidad Juarez and Tijuana among female sex workers who used opioids.^68,69^

Beyond access to syringe services programmes and opioid agonist therapy, only a few studies have characterised access to overdose prevention interventions among sex workers, despite increasing evidence that sex workers are substantially impacted by the overdose crisis.^70,71^ One study^72^ of 179 cisgender and transgender female sex workers who used drugs in Vancouver, BC, Canada, found that 97 (54%) participants had recently accessed an overdose prevention site. Another study^73^ piloting a fentanyl test strip distribution intervention among 68 cisgender female sex workers reporting past-month opioid use in Baltimore, MD, USA, reported high acceptability and uptake, with 57 (84%) participants using one or more test strips in the past month—primarily to detect the presence of fentanyl in their drugs before use.

## Health equity

The literature on mental health, SRH, and harm reduction among sex workers continues to command focus on individual factors driving inequities, with crucial gaps in data addressing systems of power, relational factors, or physiological pathways. When mapped onto Peterson and colleagues’ Health Equity Framework,^16^ the extant literature overwhelmingly focuses on substance use,^74^ mobility,^75^ internalised stigma,^76^ or other individual factors as determinants of mental health outcomes. Literature on sex workers’ SRH in the past 10 years has similarly focused on factors reflective of individual-level characteristics, including age, previous childbearing experience, and the length of time engaged in sex work.^77–80^ Within the context of literature focused on harm reduction, individual-level factors commonly explored include age, migration, and psychological distress.^81^ Across the literature, similar attention is given to relationship and network factors, including violence and coercion^82^ or number of clients,^21^ as these affect mental health, SRH, and harm reduction. A body of literature has examined how systems of power (eg, criminalisation and punitive laws and policies) shape inequitable health outcomes for sex workers, including studies assessing the mental health effects of financial security^32^ or economic recessions and COVID-19-related lockdowns.^25,30^ Within the context of SRH, fear of community stigma as a barrier to seeking health or social and support services for pregnant and parenting sex workers has been associated with a lower likelihood of contraception use^79^ and incarceration experience has been associated with a higher likelihood of unplanned pregnancy.^61^ Literature has also shown that housing instability can shape sex worker experiences with harm reduction access, including experiences of arrest and incarceration related to sex work criminalisation.^83–85^ Finally, very few studies examine physiological pathways through which sex workers’ risk for adverse health outcomes are shaped, although opportunities exist to evaluate how stress biomarkers^86^ and inflammation^87^ shape mental health, how mental health manifests as somatic symptoms,^22^ and how biological risk factors, such as genital inflammation, impact SRH outcomes more broadly.

Within the context of sex worker health, inequities manifest across interacting spheres of influence that contribute to unmet needs and suboptimal outcomes related to mental health, SRH, and substance use and harm reduction across the life course. Moreover, the current evidence reveals that access to, and uptake of, services for mental health, SRH, and drug-related harm reduction are not commensurate with the acute and chronic health-related needs expressed and experienced by sex workers globally. Addressing the complexity of the systems that foster these inequities remains central to ensuring the health and human rights of sex workers globally. However, relatively few papers interrogated heterogeneity in health outcomes for sex workers beyond individual-level behaviours or experiences. Moreover, there were few papers overall that situated differences in health outcomes within existing systems of power, including policies, laws, and institutional norms.

## Existing gaps, unmet health needs, and opportunities for research

Similar to previous studies,^9,88,89^ we found a high prevalence of depression reported across the literature, with at least one in four but in some cases four in five sex workers experiencing depressive symptoms in addition to substantial reports of PTSD, anxiety, and suicidal behaviour. When appraised alongside community literature, findings suggest that these conditions could be under-reported, as mental health symptoms often remain underdiagnosed and unaddressed given gaps in provision of accessible mental health care for sex workers.^90^ Within the SRH literature, at least one in three sex workers was estimated to experience an unmet need for contraception, reflecting larger gaps in SRH care that have been identified by sex worker communities,^8^ including a scarcity of integrated services.^91^ Literature related to the provision of harm reduction services was sparse and generally focused on sex workers communities in high-income settings despite the need for such services across multiple contexts.^62^ Overall, the extant literature communicates high unmet needs for mental health, SRH, and alcohol-related and drug-related harm reduction services among sex workers, but the coverage and scale of sex worker-oriented services in these domains remain unclear and under-interrogated in the published scholarship. This scholarly gap is likely attributed to the emergence of sex worker-focused programming in specific geographical contexts that are explicitly reflective of external funding priorities. For example, and as described in panel 3, a focus on HIV-aligned sex worker services in eastern Europe that infrequently intervene on mental health or drug-related harm reduction, rather than articulated community priorities.

Across these key health areas, literature related to sex worker health also predominately focused on cisgender women, and research focused on transgender health, cisgender men, or other gender diverse adults engaged in sex work was extremely scarce. An important number of identified papers were situated within larger HIV prevention and treatment research. Nearly all papers were cross-sectional, limiting a nuanced understanding of the evolution of health outcomes across the life course. These data also preclude any aetiological analyses to understand causal mechanisms or identify fundamental drivers of health inequities within sex work communities, particularly at a legal or policy level. Interventional papers that work to improve health by challenging existing systems of power, including the evaluation of structural change, are urgently needed to ensure optimal health outcomes for sex workers.

## A way forward

To address the unmet health needs of sex workers and inform future research priorities, we provide recommendations based on our synthesis of the literature and identification of common themes.

To tackle persistent health inequities among sex workers, a fundamental shift must be made towards sex worker community-led research and programming. Sex worker community-led research and other research that meaningfully engages sex worker communities fosters a deeper and more nuanced understanding of sex workers’ health. This Series paper prioritised sex worker community engagement at every level of conceptualisation, methodology, and interpretation. Sex workers’ right to health can only be upheld when sex workers are meaningfully involved at every level of the research process and throughout the development and implementation of health programmes, enabling sex workers to regain narrative control and correct stigmatising assumptions. With lived experience, sex workers are uniquely placed to identify the challenges their communities face and consider potential interventions to improve health. Established sex worker-led organisations provide structure and capacity building for sex workers to engage in powerful advocacy, meaningful research, and service provision.

Advancing both health and health equity for sex workers requires an intentional focus on the holistic health needs of all sex workers, including trans and gender diverse individuals, as well as cisgender men, including gay, bisexual, and other men of sexual minority. Research addressing the health of sex workers has historically centred on the experiences of cisgender women, reflecting in part the large proportion of sex workers that identify as cisgender women globally. However, although research with gender diverse sex workers has grown over the past decade, there remains little data available to disentangle the heterogeneous needs of male, trans, and other gender-diverse communities.^92^ Few studies explicitly addressed or included sex workers of all genders,^30,93,94^ limiting assessment of key health outcomes and thus overall health inequities by gender. This is particularly the case for transmasculine and non-binary sex workers, who remain vastly under-represented in the literature.^92,95,96^ In settings where punitive laws target both sex work and diverse gender expression and identity, it is crucial to understand and address intersecting forces of social oppression that negatively impact trans and nonbinary sex workers and prioritise gender affirming and appropriate care.

Over the past 10 years, research on sex workers has become inextricably linked to HIV research in a way that potentially obscures other health outcomes necessary for achieving overall health and wellbeing. Importantly, this trend reflects a high burden of HIV and other STIs among sex worker communities, necessitating a focus on research and programmes to address what remains a major health issue particularly in settings such as sub-Saharan Africa.^1,97^ However, the prominence of HIV-related research and de-prioritisation of other non-HIV and non-STI scholarship among sex workers, in part driven by global funding priorities, might have restricted scholarly inquiry into the health inequities faced by sex workers. Efforts to explore or explain health inequities among sex workers have, therefore, been traditionally anchored in HIV-related and STI-related scholarship. In the context of this Series paper, a quarter of all studies that focused on mental health, SRH, and substance use also reflected a substantive focus on HIV. This trend was pervasive across studies in how study populations were sampled, how outcomes or exposures were identified, and how the significance of findings was interpreted within the context of broader literature. Although expanding beyond HIV and other STIs is essential to address the full spectrum of sex workers’ health needs, HIV investments should be safeguarded. To prevent dilution of progress toward HIV elimination among sex workers as service and research for sex workers expands, we recommend continued monitoring of HIV outcomes to facilitate additive, and not substitutive, integration of other health services.

Research and programmes that challenge inequitable systems of power and advance sex worker community-led and based approaches are needed to better characterise and address current threats to sex workers’ health equity. Programmatically, sex worker-led efforts to mitigate and address social and structural determinants of health include advocacy work with policy makers and health-care providers, the provision of legal support services to document and challenge human rights violations, and community empowerment and mobilisation strategies to support the delivery of culturally competent and safe health care for sex workers,^98^ among others. However, the evaluation of these approaches to support a comprehensive evidence base of multilevel interventions for sex worker health is still nascent.^99^ As relatively few studies have assessed systems of power or broader structural policies and their effect on sex worker health, a crucial area for future research includes exploration of laws and policies, such as those that challenge the legal status of sex work, substance use, identity (eg, anti-LGBTQ+ laws), and migration status, across varying economic and geographical contexts. Moving forward, balanced collaborations between sex work communities and researchers can interrogate these prevailing power dynamics and generate the crucial evidence necessary to support the scalability of effective interventions aimed at enhancing the health and well being of sex workers globally.

## Supplementary Material

1

## Figures and Tables

**Figure:**
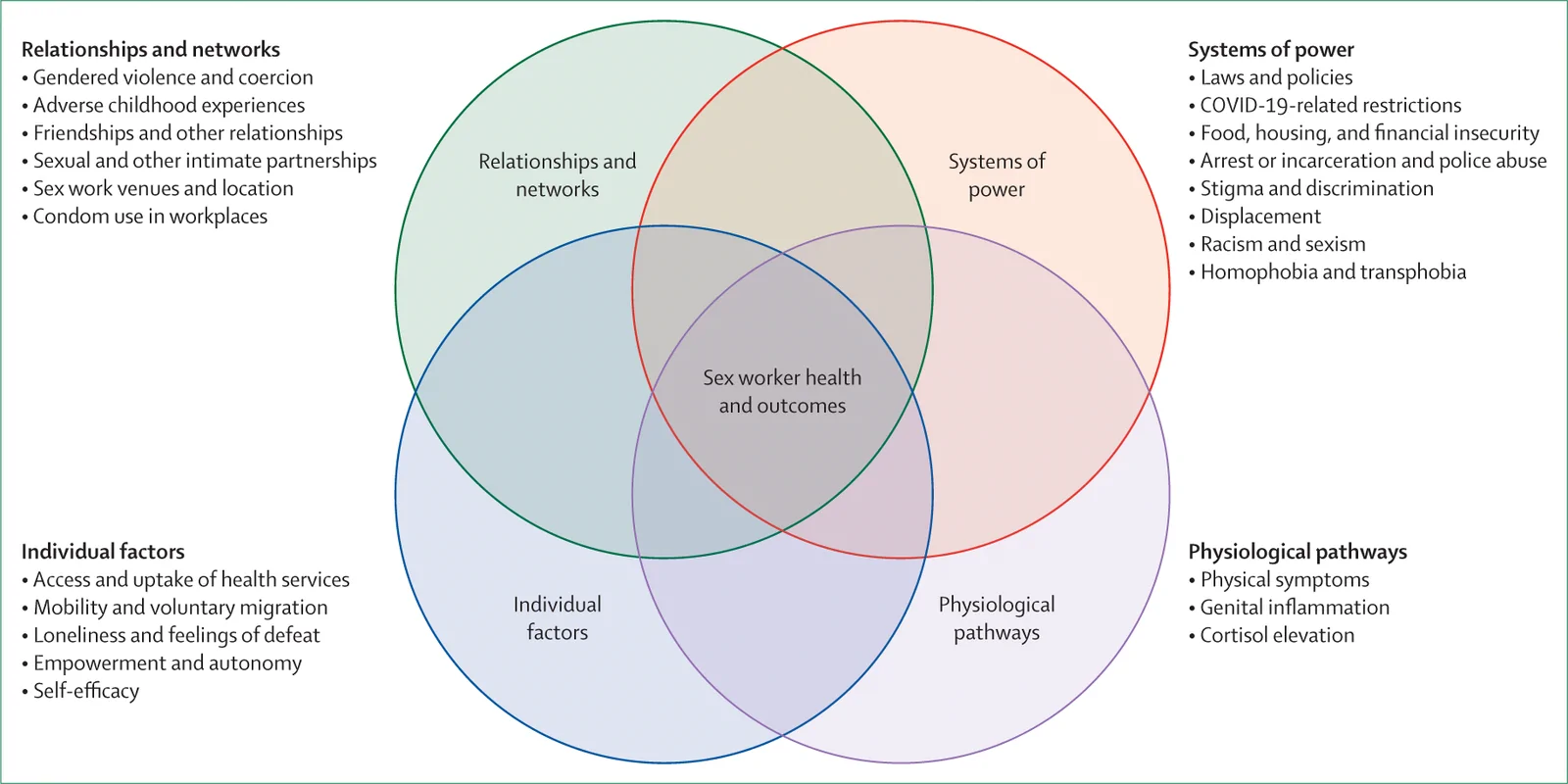
Health equity framework adapted for sex worker communities

## Key messages

- Over the past 10 years, research has documented an elevated burden of mental health symptoms among sex workers, with at least one in four and as many as four in five sex workers experiencing depression; generalised anxiety and post-traumatic stress are also elevated among sex workers, although less studied than depression
- Sexual and reproductive health literature for sex workers has predominately focused on defining gaps in access to contraception, with an estimated one in three sex workers experiencing an unmet need for contraception; however, less focus has been placed on holistic sexual and reproductive health outcomes including childbearing plans, infertility, and gender-affirming care
- Literature on harm reduction among sex workers globally is sparse, and maintains a focus on individual factors driving inequities, whereas high level factors related to systems of power and relationships and networks, are less considered
- Addressing the complexity of systems of power that foster inequities for sex workers, including criminalisation and arrest, access to and control over economic resources, and housing and the built environment remain central to ensuring the health and human rights of sex workers globally

## *Panel 1*: Accessing mental health services in the context of sex work

Testimony of a 38-year-old sex worker accessing routine health services in Ecuador.

María (pseudonym) attended a routine medical check-up, during which she was diagnosed with HIV. Instead of receiving emotional support, María was met with insensitive comments from medical staff, like don’t make any drastic decisions about this. Without guidance or empathy, she faced her diagnosis alone. Over time, the distress and stigma associated with her condition led to emotional deterioration. María began using substances as a coping mechanism and became a victim of physical and sexual violence, both by her partner and others. Despite efforts by her peers to support her, the insufficiency of professional mental health care led her to a critical depressive state and continued use of substances, culminating in suicide attempts and an eventual diagnosis of schizophrenia.

This story highlights the urgency of ensuring access to dignified, sensitive, and accessible mental health services for sex workers. Such access has the potential not only to transform individual lives but also to strengthen the social fabric by recognising and protecting the rights of a historically marginalised population. Although community support is fundamental, it cannot replace the role of a health-care system that ensures universal access to quality professional services. Without access to adequate services, María’s condition worsened, leading to severe disorientation and erratic behaviour such as wandering the streets unclothed. Timely intervention by professionals could have altered María’s trajectory, preventing the worsening of her condition and safeguarding her dignity and rights. María’s case also underscores the importance of integrating mental health services into programmes designed for sex workers. Care must go beyond basic physical health needs and adopt a sensitive and respectful approach that considers individuals’ emotional well being and unique contexts.

## *Panel 2*: Obtaining postpartum care and support in the context of sex work

Testiomy of a sex worker and social worker at a health-care facility in Guyana.

“Before I got pregnant I worked 2–3 jobs to make ends meet. After finding out about my little bundle of joy, I could only work one job. I had no knowledge of prenatal and postnatal care, labour pain, pregnancy complications, or the signs of labour. I just knew that I had to go to the clinic. The clinic does medical checkups until it’s time to give birth, but there was no preparation for my mental state of mind. Even though I’m a social worker trained to help people cope, I had my fair share of sleepless nights crying, wondering if I’ll do a good job, how will I provide, how will I buy prenatals, what will happen when I’m on leave from work (no paid leave), and my partner was struggling to find a job. The only thing that got me through those nights is that I wanted this baby for a very long time and god has finally blessed me. Now that baby is here, I’m still battling depression—why? I don’t open up easily because of my past and what I see every day. But I’m using the internet to find ways to cope and sometimes I reach out to friends for advice. Most sex workers have nowhere to turn, much less someone to reach out to.”

“Most sex workers depend on the sale of sex to survive, but while pregnant, [sex workers] cannot work. Even the acquisition of pre[-natal] and post-natal medication can prove challenging for sex workers. Mothers who are sex workers may suffer from a lack of financial and emotional support as they are caring for an infant alongside other social changes, as well as substantial financial challenges [arising from time spent away from work following birth]. With the stigma attached to sex work, some sex workers might not want to ask for assistance [even if available to support financial and medication needs]. Health-care workers need to end the stigma and discrimination. Division pushes marginalised people even further underground.”

Niki Lovisa

## *Panel 3*: Meeting the health needs of sex worker communities beyond HIV prevention and treatment

The experiences of local community-led organisations in providing health services for sex workers beyond those offered within the context of HIV prevention and treatment are crucial.

One of the few accessible and stigma-free health resources for sex workers in North Macedonia is the Health Education and Research Association (HERA)’s I Want To Know Youth Center located in Skopje. This centre offers a range of free and confidential services, including gynaecological and dermatological examinations, diagnosis and treatment of sexually transmitted infections (STIs), HIV testing, psychological counselling, and peer education. By providing essential services for sex worker communities, such as contraception, different STI tests, drug-use prevention counselling, and gynaecological checks, the centre helps reduce health inequities for sex workers who would otherwise face barriers in mainstream health-care settings. The centre serves as a crucial referral hub for sex workers and other marginalised communities, due to its longstanding reputation for offering free-of-charge and anonymous services in a non-judgemental environment and network of peer educators who refer and share information about the services available. Community-based organisations, such as STAR-STAR (the first sex workers’ collective in the Balkans), frequently refer individuals to the centre, ensuring they receive essential health services that might otherwise be inaccessible. Notably, HERA not only provides health care but also strengthens community capacity by organising training for representatives of marginalised groups and building partnerships with community-based organisations. These trainings were organised initially when the centre was first established and also focus on educating and empowering sex workers as peer educators, and equipping them to inform and guide others within their communities about the centre’s services. This peer-led outreach model has helped build trust and awareness over the years, making the centre a hotspot for the community’s health needs and a safe space for those who often face exclusion from mainstream health-care systems.

Sex workers have long faced systemic health inequities due to intersecting factors such as criminalisation, stigma, economic precarity, and insufficient access to social protections. Historically, in eastern Europe, the caucasus, and central Asia, research, funding, and programming on sex workers has focused primarily on outcomes across the HIV prevention and treatment cascade, such as HIV incidence, prevalence, and adverse service-delivery outcomes. However, the STAR Collective has broadened both research and programming to focus on other underexplored areas of health, including sexual and reproductive health, mental health, harm reduction, and access to primary health-care services. This expanded approach sheds light on the gaps in service uptake and accessibility that affect the overall well being of sex workers.

Despite the vital role of community-based organisations in addressing these health gaps, a scarcity of sustainable funding and ignorance from national stakeholders severely restricts the services they can provide. Addressing health inequities will require sustained investment in community-led services that is responsive to the diverse health needs of sex workers over the long term.

## Search strategy and selection criteria

This Series paper draws on a review of peer-reviewed epidemiological literature focused on sex worker health published over the past 10 years (2015–25). We defined sex workers as people of all genders, older than 18 years, who receive money or goods in exchange for sexual services, either regularly or occasionally, and as defined by the Global Network of Sex Worker Projects. Key health outcomes were identified a priori and were selected based on existing consolidated WHO guidelines for HIV, viral hepatitis and sexually transmitted infection prevention, diagnosis, treatment, and care for key populations. Under these guidelines, a recommended package of interventions for sex workers includes broader health interventions that extend beyond direct services for HIV. Our intention in using these guidelines was to identify related epidemiological literature and associated gaps in service to address these health outcomes with the sex worker community. We focused our search on three expansive priority areas that have been consistently understudied independent of HIV-related outcomes: mental health, sexual and reproductive health, and substance use and drug-related harm reduction service access and needs. We searched PubMed, Embase, Scopus, PsycINFO, and Cinahl for peer-reviewed publications. We report the full list of search terms for each database in the appendix (pp 3–6). Inclusion criteria comprised peer reviewed published research studies that aimed to quantitatively estimate the prevalence or incidence of a specific health outcome explicitly among sex workers, as defined above, studies published between Jan 1, 2015, and Jan 29, 2025, and studies in English, Spanish, Portuguese, or French. Exclusion criteria comprised studies that were solely qualitative in nature and excluded an intentional quantitative sampling component; studies that were systematic or scoping reviews; studies without a substantial focus on one of the topics of interest including mental health, sexual and reproductive health, and substance use and harm reduction; studies that estimated the prevalence or incidence of sex work within a different population, such as women living with HIV and women who use drugs (with the exception of studies focused on harm reduction); studies exclusively focused on transactional sex or studies in which sex work was grouped with transactional sex without disaggregation; and studies that included people younger than 18 years. We used the studies identified through the review to inform our synthesis, and then we did targeted searches to better contextualise the evidence. For all included studies, we abstracted key details and the relevant measure of occurrence for each outcome. Key associations with each measure were also reported to capture identified factors and potential determinants across multiple domains using the Health Equity Framework. Findings were contextualised against community testimonies presented in the panels, which reflect the lived experiences of sex workers, in their own words, as they navigate health services globally.
