# Supplementary material for: Health inequities faced by sex workers
Source: Lancet Public Health. Author manuscript; Available in PMC 2026 Sep 12. (PMC13570786; doi:10.1016/S2468-2667(25)00307-X)
Supplement: Supplementary file 1 [file NIHMS2205278-supplement-1.pdf]

THE LANCET
Public Health

Supplementary appendix

This appendix formed part of the original submission and has been peer reviewed. We post it as supplied by the authors.

Supplement to: Rucinski K, Roach MA, Shapiro A, et al. Health Equity for Sex Workers 1. Health inequities faced by sex workers. *Lancet Public Health* 2026; published online July 24. https://doi.org/10.1016/S2468-2667(25)00307-X.

Supplemental Methods

Search strategy, inclusion criteria, and data synthesis

This Series paper draws on a review and synthesis of peer-reviewed literature over the past ten years (2015-2025) of the epidemiologic literature addressing mental health, sexual and reproductive health (SRH), and harm reduction among sex workers globally. We defined sex workers as people of all genders over 18 years of age who receive money or goods in exchange for sexual services, either regularly or occasionally. Key health outcomes were identified *a priori* and were selected based on existing consolidated WHO guidelines for *HIV, viral hepatitis and STI prevention, diagnosis, treatment and care for key populations.* Under these Guidelines, a recommended package of interventions for sex workers includes broader health interventions that extend beyond direct services for HIV. Our intention in using these guidelines was to identify related epidemiologic literature and associated gaps in service to address these health outcomes with the sex worker community. We focused our search on three expansive priority areas, including mental health, SRH, and substance use and harm reduction. Within each of these priority areas, we also quantified the occurrence of outcomes within the context of HIV prevention and treatment studies as compared to stand alone research or programs that focused on sex worker health more broadly.

We searched PubMed, Embase, Scopus, PsycINFO, and Cinahl for peer-reviewed publications and exclude scientific abstracts. We report the full list of search terms for each database in the Supplemental Methods. Inclusion and exclusion criteria were piloted and revised through an iterative process with community authors. Inclusion criteria comprised 1) Peer reviewed published research studies that aimed to quantitatively estimate the prevalence or incidence of a specific health outcome explicitly amongst sex workers, as defined above; 2) studies published between January 1, 2015 and January 29, 2025; and 3) studies in English, Spanish, Portuguese, or French. Exclusion criteria comprised 1) Studies that were solely qualitative in nature and excluded an intentional quantitative sampling component; 2) Studies that were systematic or scoping reviews; 3) studies without a substantial focus on one of the topics of interest including mental health, sexual and reproductive health, and substance use and harm reduction; 4) studies that estimated the prevalence or incidence of sex work within a different population, such as women living with HIV and women who use drugs (with the exception of studies focused on harm reduction); 5) studies exclusively focused on transactional sex or studies in which sex work was grouped with transactional sex without disaggregation; 6) studies that include youth under the age of 18; and 7) studies published before January 1, 2015.

All identified studies were screened based on their title and abstract by two reviewers. Conflicts were resolved by a third reviewer. Selected studies then underwent a combined full text review and data abstraction process. Data extraction occurred deductively based on pre-determined categories. However, within each outcome area we allowed flexibility to inform groupings based on specific sub outcomes that emerged. For example, within the context of SRH, we included studies that reported the burden of key family planning health outcomes (e.g., proportion of sex workers using long-acting contraceptive methods) as well as studies that documented gaps in service access for family planning (e.g., received contraceptive counseling through SRH or primary health services).

After team review of the final study sample, we determined that all included studies related to substance use did not have a substantive focus on harm reduction. To amend this, we conducted a targeted harm reduction search in PubMed, using search terms outlined below. To capture studies that specifically reported harm reduction outcomes among sex workers, we also included studies previously excluded from our synthesis that comprised samples of sex workers who used drugs.

For all included studies, we abstracted key details including the year, region, and country of publication; as well as study type (e.g., observational, experimental), study design, sampling methodology, sample size, and main aim of each study. For each outcome, we extracted the relevant measure of occurrence, as well as any sub outcomes. Key associations with each measure were also reported to capture identified factors and potential determinants across multiple domains using the Health Equity Framework as proposed by Peterson *et al*.

Once all associations were abstracted, we conducted a mapping exercise whereby we aligned reported estimates to each of the relevant spheres of influence within each priority area. Factors mapped to relationships and networks included violence, adverse childhood experiences, friendships, partnership/relationship types, social support and cohesion, and financially supporting others. Those mapped to systems of power were conceptualized as larger-scale laws and policies (e.g., criminalization of sex work, restrictions related to COVID-19), as well as the resulting

downstream impacts of these laws and policies including structural determinants of health such as food insecurity, housing insecurity, and arrests. Factors mapped to physiological pathways included indicators that were biological, physical, cognitive, and psychological in nature, but not easily modifiable (e.g., hair cortisol concentration, genital inflammation) and those that reported physiological symptoms such as gastrointestinal distress. Identified indicators mapped to individual factors were widespread and included demographics, uptake or access related to health services, attitudes (e.g., loneliness, defeat, empowerment, autonomy, self-efficacy), stigma related to sex work (e.g., internal and enacted), and behavior (e.g., condom use with partners and clients, age one started selling sex), among others.

Limitations

This evidence synthesis is limited to research explicitly focused on sex workers (aged 18 and over) to align with a globally recognized definition of sex work and as recommended by community authors. As a result, studies reporting health outcomes among individuals who could be classified as sex workers—but were not defined in alignment with global definitions—may not be represented in this synthesis. Further, given we aimed to develop generalizable insights for global sex work communities, this synthesis does not include findings from studies with narrow eligibility criteria, such as those that focused explicitly on sex workers who used drugs or those that were incarcerated. Additionally, our synthetic approach required *a priori* specification of outcomes related to mental health, SRH, and substance use and harm reduction, while remaining flexible in identification of additional outcomes in these areas. While we assessed estimates of depression in this synthesis, many included studies did not report diagnostic criteria. Thus, our use of the term depression refers to both depressive symptoms as well as clinical depression. In the case that specific outcomes were not explicitly specified in our initial search (e.g., cervical cancer), we may have underestimated their relative contribution to the literature and thus underestimated potential gaps. Similarly, in the case that the gender identity of sex workers in the included studies were not explicitly mentioned by the authors, we assumed all study participants were cisgender, and thus may have underestimated the number and proportion of studies reporting health outcomes among gender diverse sex workers. Further work assessing heterogeneity in outcomes on gender is needed. Additionally, heterogeneity in both the magnitude and the quality of estimates included in this synthesis precluded more formal analytic comparisons across studies. Lastly, this synthesis did not formally include conference abstracts or grey literature as part of our search strategy. However, our findings are interpreted throughout within the context of community research, including case studies and summary reports, to ensure these critical insights are available to guide future research.

Search terms organized by database

Search terms
*10 years
Concept 1 AND (Concept 2 OR Concept 3 OR Concept 4)

PubMed: with 10 year limit: 2,855 results
Concept 1: Sex workers
"Sex Work"[Mesh] OR "Sex Workers"[MeSH] OR "sex work*"[tw] OR "sexual work*"[tw] OR prostitut*[tw] OR "commercial sex"[tw] OR "transact* sex"[tw] OR "transact* sexual*"[tw] OR "sex transact*"[tw] OR "sexual transact*"[tw] OR "SW"[tw] OR "FSW"[tw] OR "CSW"[tw] OR "sex trade*"[tw] OR "sexual trade*"[tw] OR "trading sex"[tw] OR "trading sexual*"[tw] OR "sell* sex"[tw] OR "sell* sexual*"[tw] OR "exchang* sex"[tw] OR "exchang* sexual*"[tw] OR "sex exchang*"[tw] OR "sexual exchang*"[tw]

Concept 2: Substance use
"Drug Users"[Mesh] OR "Substance Abuse, Intravenous"[Mesh] OR "substance-related disorders"[Mesh] OR "Narcotics"[Mesh] OR "drug use"[tw] OR "drug user*"[tw] OR drug abus*[tw] OR "abus* drug*"[tw] OR "substance use"[tw] OR "substance user*"[tw] OR "substance* abus*"[tw] OR "people who inject drug*"[tw] OR "people who use drug*"[tw] OR "IVDU*"[tw] OR "IDU"[tw] OR "PWUD"[tw] OR "PWID"[tw] OR "drug usage"[tw] OR "drug addict*" [tw] OR "drug depend*"[tw] OR "heroin"[tw] OR "crack"[tw] OR "cocaine"[tw] OR "meth"[tw] OR "ketamine"[tw] OR "fentanyl"[tw] OR "MDMA"[tw] OR “ecstasy"[tw] OR "mandrax"[tw] OR "cathinone"[tw] OR "whoonga"[tw] OR "nyaope"[tw] OR "opioid*"[tw] OR "barbiturate*"[tw] OR "hazardous alcohol use"[tw] OR "hazardous alcohol user*"[tw] OR "alcohol abus*"[tw] OR "abus* alcohol*"[tw] OR "binge drink*"[tw] OR "audit-c"[tw] OR "alcoholism"[tw] OR "benzodiazepine*"[tw] OR "xylazine"[tw] OR "nitazine*"[tw] OR "speedball"[tw] OR "goofball"[tw] OR "SASQ"[tw] OR "CAGE"[tw] OR "Alcohol Symptom Checklist"[tw]

Concept 2: Mental health
"Mental Disorders"[Mesh] OR "Self-injurious Behavior"[Mesh] OR "Mental Health"[Mesh] OR "Psychological Well-Being"[Mesh] OR "anxiety"[tw] OR "depression"[tw] OR "depressive*"[tw] OR "psycholog* distress"[tw] OR "trauma"[tw] OR psychotrauma*[tw] OR "psychological disorder*"[tw] OR "psychiatric disease*"[tw] OR "psychiatric disorder*"[tw] OR "psychiatric illness*"[tw] OR "mental health"[tw] OR "wellbeing"[tw] OR "mental illness"[tw] OR "self harm*"[tw] OR "self injurious"[tw] OR "self injur*"[tw] OR suicid*[tw]

Concept 2: Sexual and Reproductive health
"Reproductive Health Services"[Mesh] OR "Contraceptive Devices"[Mesh] OR "Contraceptive Agents"[Mesh] OR "Long-Acting Reversible Contraception"[Mesh] OR "Abortion, Induced"[Mesh] OR "contracepti*"[tw] OR "pregnan*"[tw] OR "birth*"[tw] OR "reproductive health"[tw] OR "prenatal care"[tw] OR "pre natal care"[tw] OR "postnatal care"[tw] OR "post natal care"[tw] OR "SRH service*"[tw] OR "abortion"[tw] OR "maternal health"[tw] OR "post partum*"[tw] OR postpartum*[tw]
****

Embase with publication year from 2015: 6,955
Concept 1: Female sex workers
'prostitution'/exp OR 'transactional sex'/exp OR ‘sex worker’/exp OR ("sex work*" OR "sexual work*" OR prostitut* OR "commercial sex" OR "transact* sex" OR "transact* sexual*" OR "sex transact*" OR "sexual transact*" OR "SW" OR "FSW" OR "CSW" OR "sex trade*" OR "sexual trade*" OR "trading sex" OR "trading sexual*" OR "sell* sex" OR "sell* sexual*" OR "exchang* sex" OR "exchang* sexual*" OR "sex exchang*" OR "sexual exchang*"):ab,ti,kw

Concept 2: Substance use
'substance abuse'/exp OR 'intravenous drug abuse'/exp OR 'drug dependence'/exp OR 'narcotic agent'/exp OR ("drug use" OR "drug user*" OR drug abus* OR "abus* drug*" OR "substance use" OR "substance user*" OR "substance* abus*" OR "people who inject drug*" OR "people who use drug*" OR "IVDU*" OR "IDU" OR "PWUD" OR "PWID" OR "drug usage" OR "drug addict*" OR "drug depend*" OR "heroin" OR "crack" OR "cocaine" OR "meth" OR "ketamine" OR "fentanyl" OR "MDMA" OR “ecstasy" OR "mandrax" OR "cathinone"

OR "whoonga" OR "nyaope" OR "opioid*" OR "barbiturate*" OR "hazardous alcohol use" OR "hazardous alcohol user*" OR "alcohol abus*" OR "abus* alcohol*" OR "binge drink*" OR "audit-c" OR "alcoholism" OR "benzodiazepine*" OR "xylazine" OR "nitazine*" OR "speedball" OR "goofball" OR "SASQ" OR "CAGE" OR "Alcohol Symptom Checklist"):ab,ti,kw

Concept 2: Mental health
'mental disease'/exp OR 'automutilation'/exp OR 'suicidal behavior'/exp OR 'mental health'/exp OR 'psychological well-being'/exp OR 'psychotrauma'/exp OR ("anxiety" OR "depression" OR "depressive*" OR "psycholog* distress" OR "trauma" OR psychotrauma* OR "psychological disorder*" OR "psychiatric disease*" OR "psychiatric disorder*" OR "psychiatric illness*" OR "mental health" OR "wellbeing" OR "mental illness" OR "self harm*" OR "self injurious" OR "self injur*" OR suicid*):ab,ti,kw

Concept 2: Sexual and Reproductive health
'health service'/exp OR 'contraceptive device'/exp OR 'contraceptive agent'/exp OR 'long-acting reversible contraception'/exp OR 'induced abortion'/exp OR ("contracepti*" OR "pregnan*" OR "birth*" OR "reproductive health" OR "prenatal care" OR "pre natal care" OR "postnatal care" OR "post natal care" OR "SRH service*" OR "abortion" OR "maternal health" OR "post partum*" OR postpartum*):ab,ti,kw

Concept 1 AND Concept 2 AND [01-01-2015]/sd
AND [2015-2025]/py

****
Cinahl (limit kw to ti, ab fields): With 2015 date limit: 1,863

Concept 1: Female sex workers
(MH "Sex Work+") OR (MH "Transactional Sex")
OR
("sex work*" OR "sexual work*" OR prostitut* OR "commercial sex" OR "transact* sex" OR "transact* sexual*" OR "sex transact*" OR "sexual transact*" OR "SW" OR "FSW" OR "CSW" OR "sex trade*" OR "sexual trade*" OR "trading sex" OR "trading sexual*" OR "sell* sex" OR "sell* sexual*" OR "exchang* sex" OR "exchang* sexual*" OR "sex exchang*" OR "sexual exchang*")

Concept 2: Substance use
(MH "Substance Abuse+") OR (MH "Substance Dependence+") OR (MH "Substance Use Disorders+") OR (MH "Substance Abuse, Intravenous") OR (MH "Persons with Substance Use Disorders+") OR (MH "Narcotics+")
OR
("drug use" OR "drug user*" OR drug abus* OR "abus* drug*" OR "substance use" OR "substance user*" OR "substance* abus*" OR "people who inject drug*" OR "people who use drug*" OR "IVDU*" OR "IDU" OR "PWUD" OR "PWID" OR "drug usage" OR "drug addict*" OR "drug depend*" OR "heroin" OR "crack" OR "cocaine" OR "meth" OR "ketamine" OR "fentanyl" OR "MDMA" OR “ecstasy" OR "mandrax" OR "cathinone" OR "whoonga" OR "nyaope" OR "opioid*" OR "barbiturate*" OR "hazardous alcohol use" OR "hazardous alcohol user*" OR "alcohol abus*" OR "abus* alcohol*" OR "binge drink*" OR "audit-c" OR "alcoholism" OR "benzodiazepine*" OR "xylazine" OR "nitazine*" OR "speedball" OR "goofball" OR "SASQ" OR "CAGE" OR "Alcohol Symptom Checklist")

Concept 2: Mental health
(MH "Psychological Trauma+") OR (MH "Mental Health") OR (MH "Mental Disorders+") OR (MH "Injuries, Self-Inflicted") OR (MH "Suicide+") OR (MH "Psychological Well-Being")
OR
("anxiety" OR "depression" OR "depressive*" OR "psycholog* distress" OR "trauma" OR psychotrauma* OR "psychological disorder*" OR "psychiatric disease*" OR "psychiatric disorder*" OR "psychiatric illness*" OR "mental health" OR "wellbeing" "mental illness" OR "self harm*" OR "self injurious" OR "self injur*" OR suicid*)

Concept 2: Sexual and Reproductive health
(MH "Reproductive Health") OR (MH "Contraceptive Agents+") OR (MH "Contraceptive Devices+") OR (MH "Long-Acting Reversible Contraceptives") OR (MH "Abortion, Induced+")

OR
("contracepti*" OR "pregnan*" OR "birth*" OR "reproductive health" OR "prenatal care" OR "pre natal care" OR "postnatal care" OR "post natal care" OR "SRH service*" OR "abortion" OR "maternal health" OR "post partum*" OR postpartum*)

****
PsycINFO (limit kw to ti, ab fields): (with date limit from 2015): 1,501

Concept 1: Female sex workers
DE "Sex Work"
OR
("sex work*" OR "sexual work*" OR prostitut* OR "commercial sex" OR "transact* sex" OR "transact* sexual*" OR "sex transact*" OR "sexual transact*" OR "SW" OR "FSW" OR "CSW" OR "sex trade*" OR "sexual trade*" OR "trading sex" OR "trading sexual*" OR "sell* sex" OR "sell* sexual*" OR "exchang* sex" OR "exchang* sexual*" OR "sex exchang*" OR "sexual exchang*")

Concept 2: Substance use
DE "Substance Related and Addictive Disorders" OR DE "Substance Use Disorder" OR DE "Alcohol Use Disorder" OR DE "Cannabis Use Disorder" OR DE "Drug Abuse" OR DE "Drug Dependency" OR DE "Inhalant Abuse" OR DE "Opioid Use Disorder" OR DE "Intravenous Drug Usage" OR DE "Narcotic Drugs" OR DE "Opiates"
OR
("drug use" OR "drug user*" OR drug abus* OR "abus* drug*" OR "substance use" OR "substance user*" OR "substance* abus*" OR "people who inject drug*" OR "people who use drug*" OR "IVDU*" OR "IDU" OR "PWUD" OR "PWID" OR "drug usage" OR "drug addict*" OR "drug depend*" OR "heroin" OR "crack" OR "cocaine" OR "meth" OR "ketamine" OR "fentanyl" OR "MDMA" OR “ecstasy" OR "mandrax" OR "cathinone" OR "whoonga" OR "nyaope" OR "opioid*" OR "barbiturate*" OR "hazardous alcohol use" OR "hazardous alcohol user*" OR "alcohol abus*" OR "abus* alcohol*" OR "binge drink*" OR "audit-c" OR "alcoholism" OR "benzodiazepine*" OR "xylazine" OR "nitazine*" OR "speedball" OR "goofball" OR "SASQ" OR "CAGE" OR "Alcohol Symptom Checklist")

Concept 2: Mental health
DE "Mental Health" OR DE "Trauma" OR DE "Stress and Trauma Related Disorders"
OR
DE "Mental Disorders" OR DE "Affective Disorders" OR DE "Anxiety Disorders" OR DE "Behavior Disorders" OR DE "Bipolar Disorder" OR DE "Borderline States" OR DE "Chronic Mental Illness" OR DE "Dissociative Disorders" OR DE "Eating Disorders" OR DE "Gender Dysphoria" OR DE "Mental Disorders due to General Medical Conditions" OR DE "Neurocognitive Disorders" OR DE "Neurodevelopmental Disorders" OR DE "Neurosis" OR DE "Obsessive Compulsive Disorder" OR DE "Paraphilias" OR DE "Personality Disorders" OR DE "Psychosis" OR DE "Serious Mental Illness" OR DE "Sleep Wake Disorders" OR DE "Somatoform Disorders" OR DE "Thought Disorders"
OR
DE "Nonsuicidal Self-Injury" OR DE "Head Banging" OR DE "Self-Inflicted Wounds" OR DE "Self-Poisoning" OR DE "Self-Inflicted Wounds" OR DE "Suicide"
OR
DE "Well Being" OR DE "Emotional Well Being"
OR
("anxiety" OR "depression" OR "depressive*" OR "psycholog* distress" OR "trauma" OR psychotrauma* OR "psychological disorder*" OR "psychiatric disease*" OR "psychiatric disorder*" OR "psychiatric illness*" OR "mental health" OR "wellbeing" "mental illness" OR "self harm*" OR "self injurious" OR "self injur*" OR suicid*)

Concept 2: Sexual and Reproductive health
(MH "Reproductive Health") OR (MH "Contraceptive Agents+") OR (MH "Contraceptive Devices+") OR (MH "Long-Acting Reversible Contraceptives") OR (MH "Abortion, Induced+")
OR

("contracepti*" OR "pregnan*" OR "birth*" OR "reproductive health" OR "prenatal care" OR "pre natal care" OR "postnatal care" OR "post natal care" OR "SRH service*" OR "abortion" OR "maternal health" OR "post partum*" OR postpartum*)
****

<u>Scopus (with date limit from 2015 (and not Medline/Embase records): 622 results</u>
<u>Concept 1: Female sex workers</u>
TITLE-ABS-KEY("sex work*" OR "sexual work*" OR prostitut* OR "commercial sex" OR "transact* sex" OR "transact* sexual*" OR "sex transact*" OR "sexual transact*" OR "SW" OR "FSW" OR "CSW" OR "sex trade*" OR "sexual trade*" OR "trading sex" OR "trading sexual*" OR "sell* sex" OR "sell* sexual*" OR "exchang* sex" OR "exchang* sexual*" OR "sex exchang*" OR "sexual exchang*")

<u>Concept 2: Substance Use</u>
TITLE-ABS-KEY ("drug use" OR "drug user*" OR drug abus* OR "abus* drug*" OR "substance use" OR "substance user*" OR "substance* abus*" OR "people who inject drug*" OR "people who use drug*" OR "IVDU*" OR "IDU" OR "PWUD" OR "PWID" OR "drug usage" OR "drug addict*" OR "drug depend*" OR "heroin" OR "crack" OR "cocaine" OR "meth" OR "ketamine" OR "fentanyl" OR "MDMA" OR “ecstasy" OR "mandrax" OR "cathinone" OR "whoonga" OR "nyaope" OR "opioid*" OR "barbiturate*" OR "hazardous alcohol use" OR "hazardous alcohol user*" OR "alcohol abus*" OR "abus* alcohol*" OR "binge drink*" OR "audit-c" OR "alcoholism" OR "benzodiazepine*" OR "xylazine" OR "nitazine*" OR "speedball" OR "goofball" OR "SASQ" OR "CAGE" OR "Alcohol Symptom Checklist")

<u>Concept 2: Mental health</u>
TITLE-ABS-KEY ("anxiety" OR "depression" OR "depressive*" OR "psycholog* distress" OR "trauma" OR psychotrauma* OR "psychological disorder*" OR "psychiatric disease*" OR "psychiatric disorder*" OR "psychiatric illness*" OR "mental health" OR "wellbeing" "mental illness" OR "self harm*" OR "self injurious" OR "self injur*" OR suicid*)

<u>Concept 2: Sexual and Reproductive health</u>
TITLE-ABS-KEY ("contracepti*" OR "pregnan*" OR "birth*" OR "reproductive health" OR "prenatal care" OR "pre natal care" OR "postnatal care" OR "post natal care" OR "SRH service*" OR "abortion" OR "maternal health" OR "post partum*" OR postpartum*)
AND (PUBYEAR > 2014)
AND NOT (INDEX(medline OR embase))

Targeted search in PubMed to identify harm reduction articles

<u>Concept 1a: Sex workers</u>
"Sex Work"[Mesh] OR "Sex Workers"[MeSH] OR "sex work*"[tw] OR "sexual work*"[tw] OR prostitut*[tw] OR "commercial sex"[tw] OR "SW"[tw] OR "FSW"[tw] OR "CSW"[tw] OR "sell* sex"[tw] OR "sell* sexual*"[tw]

<u>Concept 2a: Harm reduction</u>
"Substance Abuse Treatment"[tw] OR "Needle exchange*"[tw] OR "Needle-exchange*"[tw] OR "syringe exchange*"[tw] OR "syringe service*"[tw] OR "supervised injection*"[tw] OR "supervised consumption"[tw] OR "safe injection*"[tw] OR "safe consumption"[tw] OR "harm reduction"[tw] OR "naloxone"[tw] OR "Narcan"[tw] OR "evzio"[tw] OR "methadone"[tw] OR "suboxone"[tw] OR "naltrexone"[tw] OR "buprenorphine"[tw] OR "Opiate substitution treatment"[tw] OR "overdose prevention"[tw] OR "overdose monitoring"[tw] OR "overdose detection"[tw] OR "overdose training"[tw] OR "overdose warning"[tw] OR "drug treatment"[tw] OR "medication-assisted therapy"[tw] OR "medication-assisted treatment"[tw] OR "detox*"[tw] OR "drug rehab*"[tw] OR "safe* smoking"[tw] OR "safe* supply"[tw] OR "drug checking"[tw] OR "drug testing"[tw] OR "drug supply monitoring"[tw] OR "rapid analysis of drugs"[tw] OR "rapid chemical analysis"[tw] OR "fentanyl test*"[tw] OR "xylazine test*"[tw] OR "benzodiazepine test*"[tw]

Summary of peer-reviewed evidence

The underlying systematic review identified 177 papers published on mental health, SRH, substance use, and drug related harm reduction among sex worker communities in the last decade. The existing literature is mostly focused in countries across Africa, uses predominately cross-sectional designs, and infrequently addresses the needs of trans or male sex workers. Of the literature addressing sex workers' mental health, SRH, or harm reduction needs published in the past decade, 58 studies (33%) focused on mental health, 66 (37%) on SRH, and 63 (36%) on harm reduction and substance use. Nearly all studies used cross-sectional designs (n=156/177, 88%) and non-probability sampling methods, with only 21 (12%) using longitudinal or interventional designs reported across outcomes and regions. These studies are mainly situated in Africa (n=75/177; 42%), with a largely substantive focus on SRH outcomes, (n=39/75, 52%), including eight SRH-focused papers in Kenya and seven in South Africa. While fewer studies were identified in European and Southeast Asian countries, these data were more focused on assessing the burden of mental health outcomes (Europe: n=6/12, 50%; Southeast Asia: n=8/19, 42%). Only three studies were reported in Eastern Europe and Central Asia (Russia: 2/3; Kazakhstan: 1/3), and focused on SRH and harm reduction, respectively. Studies from the Eastern Mediterranean, Americas, and Western Pacific included a more substantial focus on substance use and its determinants (Eastern Mediterranean: 9/14, 64%, Americas: 17/40, 43%, Western Pacific: 12/23, 52%), with studies focused on harm reduction generally reported in the United States (3/11), Mexico (3/11), and Canada (4/11).

Studies ultimately included in the evidence synthesis included 123 papers published on mental health and SRH, with many papers reporting outcomes across both topics, and 11 papers published on harm reduction. These papers were broadly focused on identifying health outcomes among cisgender women, with few exceptions. Just 26 studies included transgender or gender diverse (hereafter, "trans") populations and 8 studies included cisgender men. Hereafter, the discussion refers to studies included in the evidence synthesis.

Supplemental Results

Overall trends

A total of 9,385 articles were screened after de-duplication and underwent title and abstract screening by two reviewers as part of the underlying systematic review that informed this synthesis (Figure S1). Of these articles, 435 were further assessed for eligibility through full text review. A total of 258 articles were ultimately excluded from further assessment given their misalignment with the review's inclusion criteria, including those that did not explicitly sample sex workers in their methods and design (n=28; 11%); those that reported outcomes among individuals under the age of 18 (n=61; 7%); and those that failed to broadly report an outcome within each of the three priority areas, including indicators related to mental health, sexual and reproductive health, and harm reduction (n=76; 30%), among others. Given a lack of studies on harm reduction, we included a supplemental targeted search with revised exclusion criteria resulting in a total of 11 additional articles.

Trends by year

Between 2015 and 2024 (inclusive), the total number of articles and the relative frequency of each priority area varied. The largest number of articles were identified in 2019 (n=26; 15%), and the fewest in 2024 (n=12; 7%). Articles focused on mental health comprised between 13% (n=2) and 55% (n=12) identified studies across years, those related to sexual and reproductive health between 24% (n=5) and 52% (n=14), and those related to substance use between 14% (n=3) and 56% (n=9). The three articles identified through January 2025 were excluded from any analysis of temporal trends. Of identified harm reduction studies, most were published in 2015 (n=3; 27%) and 2020 (n=3; 27%).

Distribution of studies by population

Most studies (159/177; 90%) included cisgender women in their samples, whereas a minority (13/177; 7%) included cisgender men. Sixteen studies (9%) included transgender or gender diverse (hereafter, "trans") populations, with all 16/177 (9%) including transgender women, 3/177 (2%) including transgender men, and 3/177 (2%) including nonbinary sex workers. Papers from Africa exclusively reported data from cisgender populations, with a focus on cisgender women (74/75; 99%) compared to cisgender men (4/75; 5%). Studies from the Eastern Mediterranean similarly only included cisgender women. Of studies including trans women, most came from the Western Pacific (7/23; 30%) and the Americas (6/40; 15%), whereas studies with trans men were more common in Europe (2/12; 17%). Few studies overall included nonbinary sex workers; these were from the Americas (1/40; 3%), Europe (2/12; 17%), and Western Pacific (1/23; 4%). Amongst the 11 harm reduction studies, all studies were focused on cisgender women. A majority of harm reduction studies were in the Americas (10/11; 91%), with one study in Europe (9%).

Distribution of studies by design

Of 177 included papers, 156 utilized cross-sectional study designs. There were 21 longitudinal papers captured in total. Among the 156 papers that included a cross sectional design, all used non-probability sampling methods such as network-based methods (e.g., respondent driving sampling), venue-based methods (e.g., time location sampling), or convenience sampling. Among the 21 papers that reported a longitudinal design, most leveraged existing cohorts, including those established among sex workers living with HIV (n=7). Just two included transgender women: A prospective cohort study focused on quantifying the effect of policing and social inequalities on violence and mental health in the UK; and a prospective cohort study conducted in Canada aimed at longitudinally evaluating the associations between condom use for pregnancy prevention and a novel scale that characterizes the social, policy and physical features of indoor venues and social cohesion.

Mental health

A total of 58 articles were focused on quantifying mental health outcomes among sex workers. Of these 58, articles, 10 (17%) had a primarily HIV related objective, and 40 (69%) provided an explicit definition of sex work to inform their eligibility criteria. Depression was the most reported outcome, and was captured in 49 (85%) of 58 mental health-focused papers. Twenty of these (41%) papers were from studies in Africa, 12 (25%) from the Americas, seven (14%) from Southeast Asia, five (10%) from the Western Pacific, four (8%) from Europe, and two (4%) from the Eastern Mediterranean. Anxiety was reported in 20 (35%) papers overall; eight of these (40%) were from studies in Africa, three (15%) from studies in the Americas, three (15%) from studies in South-East Asia, three (15%) from studies in Western Pacific, two (10%) from the Eastern Mediterranean Region, and two (10%) from Europe. Fifteen

of 58 mental health papers assessed PTSD (26%), including eight (53%) from studies in Africa; five (33%) from the Americas; and one (7%) from Southeast Asia and Europe. Finally, ten of the 58 mental health papers reported on suicidal ideation or behavior (17%). Seven of these (70%) were from studies in Africa, two (20%) from Southeast Asia, and one (10%) from the Americas, Europe, and the Western Pacific. Additional mental health conditions captured across these papers included bipolar disorder, personality disorders, stress and associated markers, and loneliness.

Sexual and reproductive health

A total of 66 articles were focused on quantifying SRH outcomes among sex workers. Of these 66 articles, 20 (30%) had a primarily HIV-related objective, and 51 (77%) provided a definition of sex work to inform their study inclusion criteria. Outcomes related to the use of contraception, including unmet need for contraception and the uptake of long acting reversible contraceptive (LARC) methods, were the most commonly captured outcomes and were reported among 36 (55%) of 66 papers. Twenty-three (64%) of these papers were from studies conducted in Africa, five (14%) from the Americas, four (11%) from Southeast Asia, two (6%) from the Western Pacific, and two (6%) from Europe. Fifteen total papers captured experiences of pregnancy, including unplanned or unintended pregnancy, as well as pregnancy outcomes including miscarriage and stillbirth. Ten of these (67%) were from studies in Africa, three (20%) from the Americas, and one (10%) each in Southeast Asia and the Western Pacific. An additional 12 papers reported assessments of fertility intentions or childbearing plans, of which seven (58%) were in Africa, four (33%) were in the Americas, and one (8%) was in Southeast Asia. Eighteen total papers captured HPV screening, HPV vaccine uptake, or outcomes related to cervical cancer. Of these, ten (56%) were in Africa, four (22%) were in the Americas, and five (28%) were in Southeast Asia. Additional outcomes related to SRH included prior or recent experiences of pregnancy termination, access to and uptake of antenatal or postnatal care for pregnant or mothering sex workers, and prevention of vertical transmission of HIV and/or use of safer conception methods.

Additional Considerations for Future Health Research with Sex Worker Communities

Improving mental health, SRH, and substance related harm reduction outcomes to optimize the wellbeing of sex worker communities requires addressing stigma, discrimination, and violence as persistent drivers of morbidity and mortality among sex workers globally. In this synthesis, we found that discrimination and violence were among the most common risk factors for mental health symptoms, and were frequently associated with challenges accessing SRH services. Physical manifestations of violence including injury and chronic pain have also been reported among sex workers(3), but remain understudied. Holistic approaches to sex worker wellbeing must further address upstream drivers of violence including social norms and discriminatory laws, policies and practices that enable and normalize violence toward sex workers.

Sex workers experience a complex occupational context that remains characterized by harmful and repressive laws, policies, and practices that perpetuate discrimination and health inequities in most settings globally.(6) Research and programmes pertaining to the health of sex workers are conducted against this backdrop, and may reinforce stigmatizing, inaccurate and harmful stereotypes about sex work. Research and policy discourse that has conflated sex work with sex trafficking results in harmful conclusions and ineffective policies that fail to advance sex workers' health and wellbeing.(94) Thus, engaging sex worker-led organizations and communities to set research agendas and define meaningful research questions is critical for advancing sex worker health and wellbeing, and for rebuking misperceptions around sex work that have historically perpetuated these inequities.

Life course approaches drawing on lived experiences are critical to address the unmet health needs of sex worker communities. This synthesis found much of the recent literature on sex worker health draws from cross-sectional study designs that use non-probability based sampling methods to measure health outcomes at a singular point in time. While these studies often ask about past experiences, an overreliance on cross-sectional data precludes assessment of changes in health over time, including limiting understanding of different life/developmental stages and how they reflect sex worker health. Of the few longitudinal studies that were captured in this Series paper, nearly all were cohorts situated in larger HIV-related studies(88) or from high-income countries such as the United Kingdom and Canada(28,78,79). Additionally, relatively few studies in this synthesis focus on the diverse needs of sex workers at different life stages, and literature among younger sex workers ages 18-29 is particularly nascent. Thus, addressing factors that potentially impact the health and wellbeing of sex workers necessitates higher quality study designs that include diverse perspectives from sex workers across different life stages, and particularly among younger people which may provide insights into the diverse pathways in which health outcomes are shaped by intersecting identifies and systems of oppression.

Supplemental Figure 1. PRISMA Diagram of the underlying systematic review

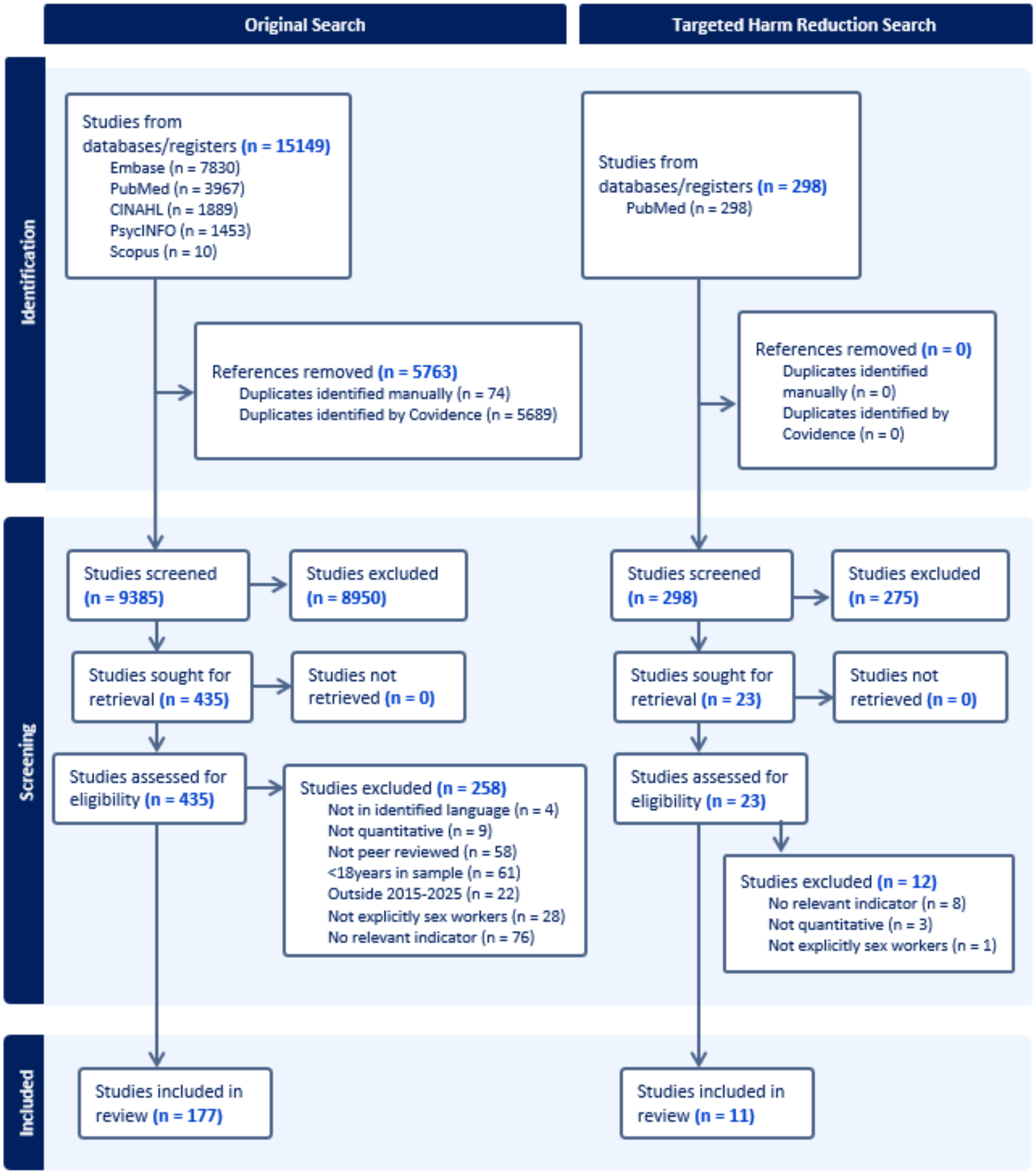

Supplemental Figure 2. Geographic distribution of 134 studies identified in the areas of mental health, sexual and reproductive health, and harm reduction identified through the underlying systematic review

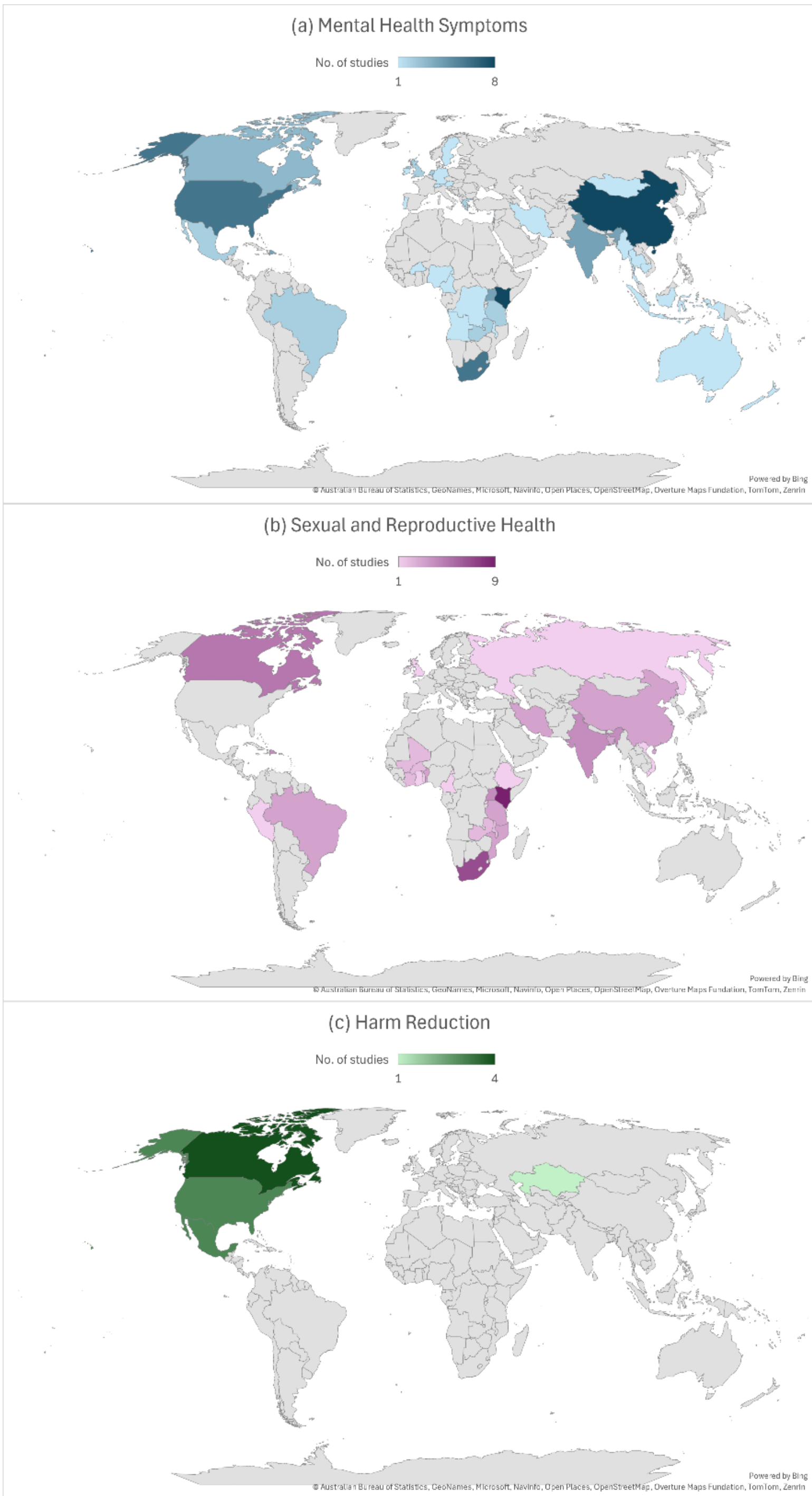

Supplemental Table 1. Reported prevalences and measurement of mental health outcomes across studies of sex worker mental health in the last decade (n=65).

Measure	Studies	Prevalence range
**Depressive symptoms**		
Beck Depression Inventory-II (BDI-II)	Semple 2019a, Semple 2019b	34.2-36.4%
Brief Symptom Inventory (BSI)	Carlson 2017, Nabunya 2021	60.4% (Nabunya: mean)
Center for Epidemiologic Studies Depression Scale (CES-D)	Rogers 2015, Krumrei-Mancuso 2016, Rael 2016, Chen 2017, Coetzee 2018, Chandler 2020, Drydakis 2021, Jewkes 2021, She 2021, Tam 2022	8.0-84.6%
Depression, Anxiety, and Stress Scale – 21 items (DASS-21)	Yasami 2023	58.5%
General Health Questionnaire – 12 items (GHQ-12)	Shen 2016	52.4%
Mini International Neuropsychiatric Interview (MINI)	Iaisuklang 2017, Ouma 2021	9-47.7%
Patient Health Questionnaire – 2 items (PHQ-2)	Patel 2015, Patel 2016, Braga 2021, Braga 2022, Bongomin 2023,Lichtwarck 2023	27.7-90.1%
Patient Health Questionnaire – 4 items (PHQ-4)	Elmes 2022	51.0%
Patient Health Questionnaire – 9 items (PHQ-9)	MacLean 2017, Poliah 2017, Wilson 2018, Abelson 2019, Chang 2019, Long 2019, Ortblad 2020, Stockton 2020, Beksinska 2021, Kerrigan 2021, Leis 2021, Lokken 2021 Rosenthal 2021, Rossouw 2021, Beksinska 2022, Tomko 2022, Wang 2022, Bhardwaj 2023, Goldenberg 2023, Grosso 2023, Beattie 2024, Panneh 2024, Wang 2024	8.0-56.8%
Zung Self-Rating Depression Scale (SDS)	Zhai 2023	32.7%
Symptom Check List Revised – 9 items	Zehnder 2019	54.0%
**Anxiety symptoms**		
Depression, Anxiety, and Stress Scale – 21 items (DASS-21)	Yasami 2023	80.0%
Generalized Anxiety Disorder – 2 item (GAD-2)	Elmes 2022, Lichtwarck 2023	32.2-51.0%
Generalized Anxiety Disorder – 7 item (GAD-7)	Beksinska 2021, Leis 2021, She 2021, Beksinska 2022, Beattie 2024, Panneh 2024	11.0-39.1%
Hospital Anxiety and Depression Scale (HADS)	Kerrigan 2021, Goldenberg 2023	11.8-34.0%
Mini International Neuropsychiatric Interview (MINI)	Iaisuklang 2017	8.0%
Zung Self-Rating Anxiety Scale (SAS)	Zhai 2023	43.1%
Structured Clinical Interview for DSM Disorders (SCID)	Ranjbar 2018	36.7%
Self-Report Questionnaire – 20 item (SRQ-20)	Poliah 2017	78.4%
**PTSD symptoms**		
Harvard Trauma Questionnaire – 17 item version	Beksinska 2021, Beksinska 2022, Beattie 2024, Panneh 2024	13.5-14.2%
Mini International Neuropsychiatric Interview (MINI)	Iaisuklang 2017	21.0%
PTSD Checklist (PCL)	Alschech 2020, Nabunya 2021	75.0% (Nabunya: mean)
PTSD Checklist for DSM-5 (PCL-5)	Park 2019, Schneider 2021, Tomko 2022	52.0-61.0%
PTSD Checklist – Civilian Version (PCL-C)	MacLean 2017	8.0%
PTSD-8	Coetzee 2018, Jewkes 2021	40.0-54.0%

Supplemental Table 2. Mental health and reproductive health studies included in the evidence synthesis.

First Author, Year	Title of Paper	Region (WHO), Country	Study Design	Sample size; Sampling Methodology	How Sex Work was Defined by the Study Authors	Gender	Mental Health Prevalence*	Reproductive Health Prevalence*
Wilson, 2018	A prospective cohort study of fertility desire, unprotected sex, and detectable viral load in HIV-positive female sex workers in Mombasa, Kenya	African Region, Kenya	Longitudinal, Observational	255; Venue-based Sampling	Defined as women ≥ 18 years old and reported ever exchange of sex for cash or in-kind payment	Cisgender women	Depression 28%; PTSD 14%	Fertility intentions† 26%
Rosenthal, 2021	A Prospective Study of Depressive Symptoms, Condomless Sex, and HIV Viral Load in HIV-Positive Female Sex Workers in Kenya	African Region, Kenya	Longitudinal, Observational	369; N/A	Not defined	Cisgender women	Depression 27%	
Ross, 2021	Access to sexual and reproductive health care among young adult sex workers in Toronto, Ontario: a mixed-methods study	Region of the Americas, Canada	Cross-sectional, Observational	112; Convenience Sampling	Defined as the exchange of sexual services for money, services or goods	Cisgender women, Cisgender men, Transgender women, Transgender men, Nonbinary/Other		
Schwartz, 2015	An urgent need for integration of family planning services into HIV care the high burden of unplanned pregnancy, termination of pregnancy, and limited contraception use among female sex workers in Côte d'Ivoire	African Region, Côte d'Ivoire	Cross-sectional, Observational	466; Respondent-driven Sampling	Defined as those who reported >50% of income from sex work over the past 12 months	Cisgender women, Transgender men		Termination 64%; Antenatal and/or postnatal care 85%
Parmley, 2019	Antenatal care presentation and engagement in the context of sex work: exploring barriers to care for sex worker mothers in South Africa	African Region, South Africa	Cross-sectional, Observational	410; Respondent-driven Sampling	Defined as cisgender women ≥18 years who made the majority of their income from exchanging sex for money	Cisgender women		Termination 65%; Antenatal and/or postnatal care 90%
Beattie, 2024	Are Violence, Harmful Alcohol/Substance Use and Poor Mental Health Associated with increased Genital inflammation?: A Longitudinal Cohort Study with HIV-Negative Female Sex Workers in Nairobi, Kenya.	African Region, Kenya	Longitudinal, Observational	526; simple random sampling	Not defined	Cisgender women	Depression and/or anxiety 54%	
Stockton, 2020	Associations among Experienced and internalized Stigma, Social Support, and Depression among Male and Female Sex Workers in Kenya	African Region, Kenya	Cross-sectional, Observational	729; snowball sampling	Defined as those who earned a significant part of their income over the last three months from sex work	Cisgender women, Cisgender men, Transgender women, Transgender men, Nonbinary/Other	Depression 34%	
Menon, 2016	Associations between vaginal infections and potential high-risk and high-risk Human Papillomavirus (HPV) genotypes in female sex workers (FSWs) in western Kenya	African Region, Kenya	Cross-sectional, Observational	616; Snowball Sampling	Defined as those who are engaging in transactional sex in exchange for money, goods, services, or drugs in the last 3 months	Cisgender women		HPV infection 58%

Panneh, 2024	Associations of Hair Cortisol Levels with Violence, Poor Mental Health, and Harmful Alcohol and Other Substance Use among Female Sex Workers in Nairobi, Kenya	African Region, Kenya	Cross-sectional, Observational	425; simple random sampling	Not defined	Cisgender women	Depression 24%; Anxiety 12%; PTSD 14%; Suicidal ideation and/or suicide attempt 11%	
Aharon, 2017	Barriers to follow-up for abnormal papanicolaou smears among female sex workers in Lima, Peru	Region of the Americas, Peru	Cross-sectional, Observational	97; Convenience Sampling	Not defined	Cisgender women		Cervical cancer screening 27%
LaFort, 2016	Barriers to HIV and sexual and reproductive health care for female sex workers in Tete, Mozambique: results from a cross-sectional survey and focus group discussions	African Region, Mozambique	Cross-sectional, Observational	311; Respondent-driven Sampling	Defined as women who engage in sex work, either as a main occupation (full-time FSWs) or as a secondary activity (occasional FSWs).	Cisgender women		
Wang, 2022	Biomedical contraceptives and their association with condom use consistency among female sex workers in China: Results from a national cross-sectional study	Western Pacific Region, China	Cross-sectional, Observational	1229; Venue-based Sampling	Defined as those who had exchanged sex for money or goods at least once in the past 3 months	Cisgender women		
Puri, 2017	Burden and Correlates of Mental Health Diagnoses among Sex Workers in an Urban Setting	Region of the Americas, Canada	Cross-sectional, Observational	692; time location sampling	Defined as those who self-identify as a woman/female, including transgender individuals who identify as women (male-to-female); having exchanged sex for money within the last 30 days;	Cisgender women, Transgender women	Depression 35%; Anxiety 20%; PTSD 13%	
Richards, 2018	Cervical cancer screening among transactional female sex workers in the Dominican Republic	Region of the Americas, Dominican Republic	Cross-sectional, Observational	144; Venue-based Sampling	Defined as women of ages 18–60 years who self-identified as sex workers and had exchanged sexual services for payment or gifts within the last six months	Cisgender women		HPV infection 43%; Cervical cancer screening 77%
Adams, 2019	Cervical Human Papillomavirus Prevalence (HPV), genotypes, and associated risk factors among female sex workers (FSWs) in greater Accra, Ghana	African Region, Ghana	Cross-sectional, Observational	100; Snowball Sampling	Not defined	Cisgender women		HPV infection 26%
Schneider, 2021	Conceptualizing Overdose Trauma: The Relationships between Experiencing and Witnessing Overdoses with Ptsd Symptoms among Street-Recruited Female Sex Workers in Baltimore, Maryland	Region of the Americas, United States	Cross-sectional, Experimental	380; venue based sampling	Defined as those who traded sex for money, goods, or drugs three or more times in the past 3 months.	Cisgender women	PTSD 52%	
Mbita, 2019	Consistent condom use and dual protection among female sex workers (FSWs): surveillance findings from a large-scale, community-based combination HIV prevention program in Tanzania	African Region, Tanzania	Cross-sectional, Observational	119468; Convenience Sampling	Defined as those who are exchanging sex for cash or goods as her primary and current source of income (over half of her monthly income)	Cisgender women		

Martin, 2015	Contraceptive use among female sex workers in three Russian cities	European Region, Russia	Cross-sectional, Observational	708; Respondent-driven Sampling	Defined as women aged 18 years and older who reported trading sex for money, drugs, or shelter in the previous 3 months.	Cisgender women		
Chanda, 2017	Contraceptive use and unplanned pregnancy among female sex workers in Zambia	African Region, Zambia	Cross-sectional, Observational	946; convenience sampling	Not defined	Cisgender women		Termination 48%
Alschech, 2020	Contributors To Posttraumatic Stress Symptoms in Women Sex Workers	Region of the Americas, United States; Canada	Cross-sectional, Observational	314; convenience sampling	Defined as self identified women who sell sex who advertise online	Cisgender women, Transgender women	PTSD 75%	
Patel, 2015	Correlates of Mental Depression among Female Sex Workers in Southern India	South-East Asia Region, India	Cross-sectional, Observational	1986; cluster sampling	Defined as women 18 years or older who had sex in exchange for cash/kind in the past 1 month were referred to as FSWs	Cisgender women	Depression 39%	
Twizelimana, 2020	Correlates of pregnancy among female sex workers (FSWs) in semi urban Blantyre, Malawi	African Region, Malawi	Cross-sectional, Observational	200; Snowball Sampling	Defined as exchanged sex for money or goods	Cisgender women		
Zhai, 2023	Correlation Analysis Between Sleep Quality and the Mental Health Status of Female Sex Workers during the Covid-19 Pandemic in Hubei Province	Western Pacific Region, China	Cross-sectional, Observational	826; cluster sampling	Not defined	Cisgender women	Depression 33%; Anxiety 43%	
Lu, 2024	Correlation Between Social Support and HPV Vaccination Behavior of Female Sex Workers in Entertainment Venues in a Region of Guangxi, China	Western Pacific Region, China	Cross-sectional, Experimental	921; Purposive Sampling	Defined as female sex workers (FSWs) refer to women who have sex with males in order to obtain material rewards, such as money.	Cisgender women		
Park, 2019	Cumulative Violence and PTSD Symptom Severity among Urban Street-Based Female Sex Workers	Region of the Americas, United States	Cross-sectional, Observational	220; target sampling	Defined as those who sold or traded oral, vaginal, or anal sex "for money or things like food, drugs, or favors"; picked up clients on the street or at public places ≥3 times in the past 3 months	Cisgender women	PTSD 61%	
Reza-Paul, 2019	Delivering community-led integrated HIV and sexual and reproductive health services for sex workers: A mixed methods evaluation of the DIFFER study in Mysore, South India	South-East Asia Region, India	Cross-sectional, Observational	415; Respondent-driven Sampling	Not defined	Cisgender women		Termination 88%; Cervical cancer screening 12%
Shen, 2016	Depression and HIV Risk Behaviors among Female Sex Workers in Guangdong, China: A Multicenter Cross-Sectional Study	South-East Asia Region, China	Cross-sectional, Observational	653; venue based convenience sampling	Not defined	Cisgender women	Depression 52%	
Rael, 2016	Depression and Key Associated Factors in Female Sex Workers and Women Living with HIV/Aids in the Dominican Republic	Region of the Americas, Dominican Republic	Cross-sectional, Observational	349; venue based sampling	Not defined	Cisgender women	Depression 70%	

Coetzee, 2018	Depression and Post Traumatic Stress amongst Female Sex Workers in Soweto, South Africa: A Cross Sectional, Respondent Driven Sample	African Region, South Africa	Cross-sectional, Observational	508; respondent driven sampling	Defined as those who sold sex in Soweto at the time of the study	Cisgender women	Depression 69%; PTSD 40%	
Poliah, 2017	Depression, Anxiety Symptoms and Substance Use amongst Sex Workers Attending a Non-Governmental Organisation in Kwazulu-Natal, South Africa	African Region, South Africa	Cross-sectional, Observational	155; convenience sampling	Not defined	Cisgender women, Cisgender men, Transgender women, Transgender men, Nonbinary/Other	Depression 81%; Anxiety 78%; Suicidal ideation 40%; Suicide attempt 30.3%	
Sabo, 2019	Does bacterial vaginosis modify the effect of hormonal contraception on HIV seroconversion: a prospective cohort study	African Region, Kenya	Longitudinal, Observational	1985; Consecutive sampling	Defined as women aged 18-50 years and reporting exchange of sex for cash or in-kind payment	Cisgender women		
Bongomin, 2023	Dual contraceptive use and associated factors among female sex workers in Gulu City, Uganda in 2023	African Region, Uganda	Cross-sectional, Observational	314; Consecutive sampling	Not defined	Cisgender women	Depression 90%	Termination 47%
Lichtwarck, 2023	Early Disengagement From HIV Pre-Exposure Prophylaxis Services and Associated Factors among Female Sex Workers in Dar Es Salaam, Tanzania: A Socioecological Approach	African Region, Tanzania	Cross-sectional, Observational	470; respondent-driven sampling	Defined as those having sold sex for money or goods in the last 3 months before study recruitment	Cisgender women	Depression and/or anxiety 32%	
Lafort, 2018	Effect of a ‘diagonal’ intervention on uptake of HIV and reproductive health services by female sex workers in three sub-Saharan African cities	African Region, South Africa	Longitudinal, Observational	Durban: 400 baseline, 400 endline; Tete: 311 baseline, 404 endline; Mombasa: 400 baseline, 403 endline; Respondent-driven Sampling	Defined as those who have had sex in exchange for money or goods at least three times in the last 6 months	Cisgender women		Cervical cancer screening 0%-32%
Elmes, 2022	Effect of Police Enforcement and Extreme Social inequalities on Violence and Mental Health among Women Who Sell Sex: Findings from a Cohort Study in London, UK	European Region, England	Longitudinal, Observational	197; time-location sampling, convenience sampling in clinics and using snowball sampling	Defined as cisgender and transgender women who provided in-person sexual services in the previous 3 months in East London (later expanded to all London for off-street sex workers only) and 18 years or older	Cisgender women, Transgender women, Nonbinary/Other	Depression and/or anxiety 51%	
Ngo, 2023	Effectiveness of mHealth intervention on safe abortion knowledge and perceived barriers to safe abortion services among female sex workers in Vietnam	Western Pacific Region, Vietnam	Longitudinal, Quasi-experimental	251; respondent driven sampling (online)	Defined as self identified as FSW/woman	Cisgender women		Termination 93%
Cange, 2019	Effects of Traumatic Events on Sex Workers’ Mental Health and Suicide Intentions in Burkina Faso: A Trauma-informed Approach	African Region, Burkina Faso	Cross-sectional, Observational	696; respondent driven sampling	Defined as those who reported selling sex within the past 12 months with the majority of income coming from sex work	Cisgender women	Depression 42%; Suicidal ideation 21%	
Srivatsan, 2019	Engagement in antiretroviral treatment and modern contraceptive methods	African Region, Lesotho	Cross-sectional, Observational	744; RDS	Defined as reported sex work as their primary source of income for the past 6 months	Cisgender women		Fertility intentions† 40%

	among female sex workers (FSWs) living with HIV in Lesotho							
Rwema, 2019	Evaluating the vertical HIV transmission risks among South African female sex workers; have we forgotten PMTCT in their HIV programming?	African Region, South Africa	Cross-sectional, Observational	410; Respondent-driven Sampling	Defined as cisgender women who were at least 18 years old, reporting sex work as their main source of income in the year preceding the study	Cisgender women		
Teixeira, 2015	Exploratory Study on the Prevalence of Suicidal Behavior, Mental Health, and Social Support in Female Street Sex Workers in Porto, Portugal	European Region, Portugal	Cross-sectional, Observational	52; convenience sampling	Not defined	Cisgender women	Depression and/or anxiety 56%; Suicidal ideation 46%; Suicide attempt 44%	
Rossouw, 2021	Exploring the Association between Depression and Social and Biobehavioral HIV Risk Factors among Female Sex Workers in Nelson Mandela Bay Municipality, South Africa	African Region, South Africa	Cross-sectional, Observational	410; Respondent driven sampling	Defined as current cisgender women aged 18 and older making the majority of their income through sex work	Cisgender women	Depression 30%	
Hart, 2022	Exploring the Relationships Between Internalized Stigma, Loneliness, and Mental Well-Being among Sex Workers	European Region, Western Pacific Region, Australia; New Zealand; United Kingdom; China; Ireland; Austria; Germany; Greeze; Netherlands; Sweden	Cross-sectional, Observational	56; convenience sampling	Defined as those who identify as full-service sex workers, indicating provision of in-person sex services	Cisgender women, Cisgender men, Transgender women, Transgender men		
Ouma, 2022	Factors associated with the uptake of long-acting reversible contraception among female sex workers in post-conflict Northern Uganda: a cross-sectional study	African Region, Uganda	Cross-sectional, Observational	280; Simple Random Sampling	Defined as females aged 18 years and above who had been active in sex work within 6 months before data collection	Cisgender women, Transgender men		Termination 37%
Opito, 2025	Factors associated with uptake and acceptability of cervical cancer screening among female sex workers in Northeastern Uganda: A cross-sectional study	African Region, Uganda	Cross-sectional, Observational	423; Consecutive Sampling	Not defined	Cisgender women		Cervical cancer screening 33%
Slim, 2020	Factors Influencing Women's Sex Work in a Lebanese Sample: Results of a Case-Control Study	Eastern Mediterranean Region, Lebanon	Cross-sectional, Observational	60; convenience sampling	Defined as women in prison who identified as sex worker	Cisgender women		
Cernigliaro, 2018	Factors related to fertility desire among female sex workers living with HIV in the Dominican Republic	Region of the Americas, Dominican Republic	Cross-sectional, Observational	247; Respondent-driven Sampling; Convenience Sampling	Defined as women who reported having exchanged sex for money in the last month.	Cisgender women		Fertility intentions† 28%

Cernigliaro, 2016	Factors related to pregnancy among female sex workers (FSWs) living with HIV in the Dominican Republic	Region of the Americas, Dominican Republic	Cross-sectional, Observational	268; Purposive sampling; Snowball Sampling	Defined as the exchange of sex for money	Cisgender women		Fertility intentions† 26%
Beckhan, 2021	Family planning use and correlates among female sex workers in a community empowerment HIV prevention intervention in Iringa, Tanzania: a case for tailored programming	African Region, Tanzania	Cross-sectional, Experimental	339; Time-location sampling	Not defined	Cisgender women		Fertility intentions† 22%; Antenatal and/or postnatal care 85%
Chang, 2019	Feelings of Entrapment and Defeat Mediate the Association Between Self-Esteem and Depression among Transgender Women Sex Workers in China	Western Pacific Region, China	Cross-sectional, Observational	198; Snowball sampling	Defined as self-identified transgender women sex workers who had intercourse with ≥1 male client in the last 3 months	Transgender women	Depression 25%	
Ingabire, 2019	Female sex workers in Kigali, Rwanda: a key population at risk of HIV, sexually transmitted infections, and unplanned pregnancy	African Region, Rwanda	Cross-sectional, Observational	1168; Venue-based Sampling;	Defined as any woman exchanging sexual favors for money	Cisgender women		
Bitty-Anderson, 2024	Gynecological health care services utilization and violence among female sex workers in Togo in 2021	African Region, Togo	Cross-sectional, Observational	447; Snowball sampling	Defined as women engaging in transactional sex at least in the past 6 months; having sex in exchange for money or goods with clients as opposed to emotional partners or boyfriends);	Cisgender women		Fertility intentions† 59%; Termination 40%; Cervical cancer screening 8%
Semple, 2019	Hazardous Alcohol Consumption Moderates the Relationship Between Safer Sex Maintenance Strategies and Condomless Sex with Clients among Female Sex Workers in Mexico	Region of the Americas, Mexico	Longitudinal, Observational	602; time location sampling	Defined as cisgender female who were at least 18 years of age; self-identify as a female sex worker; report having traded sex for drugs, money, shelter, or other material benefit in the previous month;	Cisgender women	Depression 34%	
Braga, 2022	Health Vulnerabilities in Female Sex Workers in Brazil, 2016	Region of the Americas, Brazil	Cross-sectional, Observational	4245; respondent driven sampling	Defined as those who reported working as a sex worker in one of the study cities, and who had at least 1 sexual intercourse in exchange for money in the past 4 months	Cisgender women	Depression 28%	Antenatal and/or postnatal care 86%; Cervical cancer screening 62%
Lafort, 2018	Impact of a "diagonal" intervention on uptake of sexual and reproductive health services by female sex workers (FSWs) in Mozambique: a mixed-methods implementation study	African Region, Mozambique	Cross-sectional, Quasi-experimental	Pre-intervention: 311; Post-intervention: 404; Respondent-driven Sampling	Defined as women aged 18 years or older who were actively engaging in sex work within the study area at the time of the survey.	Cisgender women		Cervical cancer screening 0%
She, 2021	Impact of Minority Stress and Poor Mental Health on Sexual Risk Behaviors among Transgender Women Sex Workers in Shenyang, China	Western Pacific Region, China	Cross-sectional, Observational	204; snowball sampling	Defined as those who have had anal intercourse with ≥ 1 male commercial sex client (last three months)	Transgender women	Anxiety 51%	

Lokken, 2021	Incidence and correlates of pregnancy in HIV-positive Kenyan sex workers	African Region, Kenya	Longitudinal, Observational	279; Convenience Sampling	Defined as women who enter and leave sex work	Cisgender women	Depression 29%	Fertility intentions† 29%
Khezri, 2020	Induced abortion and associated factors among female sex workers in Iran	Eastern Mediterranean Region, Iran	Cross-sectional, Observational	1335; Convenience Sampling	Defined as having sex in exchange for livelihood (i.e., money, goods, services or drugs) with more than one client within the 12 month period before the interview	Cisgender women		Termination 47%
Jewkes, 2021	Intersections of Sex Work, Mental Ill-Health, IPV and Other Violence Experienced By Female Sex Workers: Findings From A Cross-Sectional Community-Centric National Study in South Africa	African Region, South Africa	Cross-sectional, Observational	3005; respondent driven sampling	Defined as those who voluntarily sold or transacted in sex for financial gain (not necessarily paid in cash) in the past six months.	Cisgender women	Depression 53%; PTSD 54%	
Leis, 2021	Intimate Partner and Client-Perpetrated Violence Are Associated with Reduced HIV Pre-Exposure Prophylaxis (Prep) Uptake, Depression and Generalized Anxiety in A Cross-Sectional Study of Female Sex Workers From Nairobi, Kenya	African Region, Kenya	Cross-sectional, Observational	220; convenience sampling	Not defined	Cisgender women	Depression 57%; Anxiety 39%	
Yam, 2016	Kenyan female sex workers' use of female-controlled nonbarrier modern contraception: do they use condoms less consistently?	African Region, Kenya	Cross-sectional, Observational	596; Respondent-driven Sampling	Defined as women ages 18 years and older who reported selling sex for money, drugs or goods to a man in the past 3months	Cisgender women		Termination 19%
Ortblad, 2020	Knowledge of HIV Status Is Associated with a Decrease in the Severity of Depressive Symptoms among Female Sex Workers in Uganda and Zambia	African Region, Uganda; Zambia	Longitudinal, Experimental	1925; convenience sampling	Defined as those who exchanged money or goods for sex in the past month	Cisgender women	Depression 43%-46%	
Argaw, 2022	Knowledge, and practice of cervical cancer prevention and associated factors among commercial sex workers in Shashemene Town, West Arsi, Oromia Region, Ethiopia	African Region, Ethiopia	Cross-sectional, Observational	385; Systematic random sampling	Defined as commercial sex workers who are women	Cisgender women		Cervical cancer screening 20%
Qayum, 2022	Knowledge, attitude and practice of commercial sex workers regarding cervical cancer and its screening, Daulatdia Brothel, Rajbari District, Bangladesh, 2020-2021	South-East Asia Region, Bangladesh	Cross-sectional, Observational	400; Convenience Sampling	Defined as women living in the Daulatdia brothel for more than 3 months	Cisgender women		
Sitonga, 2023	Knowledge, attitudes, and practices of sex workers of three South African towns towards female condom use and contraceptives	African Region, South Africa	Cross-sectional, Observational	69; Convenience Sampling	Not defined	Cisgender women		
Karamouzian, 2016	Lifetime abortion of female sex workers in Iran: findings of a national bio-behavioural survey in 2010	Eastern Mediterranean Region, Iran	Cross-sectional, Observational	863; Convenience Sampling from venues	Defined as those who had exchanged sex for money, drugs, or goods at least once during the previous year; and had a history of practicing sex work for at least 6 months	Cisgender women		Termination 35%

Abelson, 2019	Lifetime Experiences of Gender-Based Violence, Depression and Condom Use among Female Sex Workers in Cameroon	African Region, Cameroon	Cross-sectional, Observational	2165; respondent driven sampling	Defined as women who practiced sex work within the past 3 months and reported sex work as their principal source of income	Cisgender women	Depression 50%	
Wang, 2024	Longitudinal Analysis of the Role of Mindfulness on HIV Stigma, Depression, Substance Use and HIV Outcomes among Female Sex Workers Living with HIV in the Dominican Republic	Region of the Americas, Dominican Republic	Longitudinal, Observational	240; convenience sampling	Defined as those who are exchanging sex for money in the last month	Cisgender women	Depression 70%	
Beksinska, 2022	Longitudinal Experiences and Risk Factors For Common Mental Health Problems and Suicidal Behaviours among Female Sex Workers in Nairobi, Kenya	African Region, Kenya	Longitudinal, Observational	1003 at baseline; 366 at midline; 877 at endline; simple random sampling	Defined as those who exchanged money for sex or accepted goods as payment	Cisgender women	Depression 23%; Anxiety 11%; PTSD 14%; Suicidal ideation 10%; Suicide attempts 3%	
Perrault-Sullivan, 2023	Longitudinal study of pregnancy intention and its association with pregnancy occurrence among female sex workers in Benin and Mali	African Region, Benin; Mali	Longitudinal, Observational	600; Convenience Sampling	Not defined	Cisgender women		Fertility intentions† 16%
Semple, 2019	Maternal Role Strain and Depressive Symptoms among Female Sex Workers in Mexico: the Moderating Role of Sex Work Venue	Region of the Americas, Mexico	Cross-sectional, Observational	426; time location sampling	Defined as cisgender female, at least 18 years of age, who self-identifies as a female sex worker, and reported having traded sex for drugs, money, shelter or other material benefit in the previous month	Cisgender women	Depression 36%	
Tomko, 2022	Mental Health and HIV Risk Differs By Co-Occurring Structural Vulnerabilities among Women Who Sell Sex	Region of the Americas, United States	Cross-sectional, Observational	385; venue based sampling	Defined as those who sold or traded sex at least three times in the past three months	Cisgender women		
She, 2021	Mental Health Service Utilisation among Transgender Women Sex Workers Who Are At Risk of Mental Health Problems in Shenyang, China: An Application of Minority Stress Theory	Western Pacific Region, China	Cross-sectional, Observational	199; convenience sampling	Defined as those who reported anal intercourse with ≥1 male commercial sex client (last 3 months)	Transgender women	Depression 71%-90%; Anxiety 69%-79%; Suicidal ideation 24%-27%	
Ranjbar, 2018	Mental Health Status among Female Sex Workers in Tabriz, Iran	Eastern Mediterranean Region, Iran	Cross-sectional, Observational	48; simple random sampling	Defined as those who are registered as a sex worker at an NGO	Cisgender women		
Kanayama, 2022	Mental Health Status of Female Sex Workers Exposed To Violence in Yangon, Myanmar	South-East Asia Region, Myanmar	Cross-sectional, Observational	403; snowball sampling	Not defined	Cisgender women		
Kerrigan, 2021	Mindfulness, Mental Health and HIV Outcomes among Female Sex Workers in the Dominican Republic and Tanzania	African Region, Region of the Americas, Dominican Republic and Tanzania	Cross-sectional, Observational	408; Dominican Republic: snowball sampling ; Tanzania: time location sampling	Not defined	Cisgender women	Depression 11-38%; Anxiety 12%-18%	

Tam, 2022	Mindfulness, Psychological Distress, and Somatic Symptoms among Women Engaged in Sex Work in China	Western Pacific Region, China	Cross-sectional, Observational	410; venue based sampling	Defined as women who provided commercial sex	Cisgender women	Depression 8%	Unmet need for contraception 35%
Papworth, 2015	Mothers who sells sex: A potential paradigm for integrated HIV, sexual, and reproductive health interventions among women at high risk of HIV in Burkina Faso	African Region, Burkina Faso	Cross-sectional, Observational	696; Respondent-driven Sampling	Defined as women who obtained the majority of their annual income from commercial sex work	Cisgender women		
Chen, 2017	Negative Trust and Depression among Female Sex Workers in Western China: The Mediating Role of Thwarted Belongingness	South-East Asia Region, China	Cross-sectional, Observational	457; convenience sampling and snowball sampling	Not defined	Cisgender women	Depression 41%	
Reed, 2015	Non-barrier contraceptive use and relation to condom use behaviour by partner type among female sex workers in Andhra Pradesh, India	South-East Asia Region, India	Cross-sectional, Observational	2197; Respondent-driven Sampling	Defined as females aged ≥18 years who reported having sex in exchange for money in the year prior to the survey.	Cisgender women, Transgender men		Fertility intentions† 29%
Sullivan, 2020	Overview and factors associated with pregnancies and abortions occurring in sex workers in Benin	African Region, Benin	Cross-sectional, Observational	866; Venue-based Sampling	Defined as selling sex for money or goods at the time of the study	Cisgender women		Termination 11%
Cernigliaro, 2019	Patient-provider communication about pregnancy and HIV among female sex workers living with HIV in Santo Domingo, Dominican Republic	Region of the Americas, Dominican Republic	Cross-sectional, Experimental	253; Convenience Sampling	Defined as having exchanged sex for money in the last month	Cisgender women		Fertility intentions† 64%; Termination 40%
Yasami, 2023	Poverty, Psychological Distress, and Suicidality among Gay Men and Transgender Women Sex Workers During the Covid-19 Pandemic in Phuket, Thailand	South-East Asia Region, Thailand	Cross-sectional, Observational	270; snowball sampling	Defined as Thai gay men and transgender women who engage in sex work or provide services in the sex tourism industry	Cisgender men, Transgender women	Depression 87%; Anxiety 80%	Termination 50%
Nabunya, 2021	Predictors of Depressive Symptoms and Post Traumatic Stress Disorder among Women Engaged in Commercial Sex Work in Southern Uganda	African Region, Uganda	Cross-sectional, Experimental	542; respondent driven sampling and snowball sampling	Defined as engaging in sex acts in exchange for pay	Cisgender women		Termination 47%
Duff, 2016	Pregnancy intentions among female sex workers: recognizing their rights and wants as mothers.	Region of the Americas, Canada	Cross-sectional, Observational	510; Time-location Sampling (online)	Defined as female (inclusive of transgender) and having exchanged sex for money within the past month	Cisgender women, Transgender men		Fertility intentions† 28%
Rao, 2016	Pregnancy intentions and safer pregnancy knowledge among female sex workers in Port Elizabeth, South Africa	African Region, South Africa	Cross-sectional, Observational	410; Respondent-driven Sampling	Defined as assigned female sex at birth, reported practicing sex work within the last year as a main source of income	Cisgender women, Transgender men		Fertility intentions† 48%; Termination 11%
Lowe, 2019	Pregnancy prevention and contraceptive preferences of online sex workers in the UK	European Region, England	Cross-sectional, Observational	67; Convenience Sampling	Defined as advertised on the internet and had sexual contact with clients, participants were purposefully chosen to cover a range of online sex work experiences, including those managing others (i.e., an escort	Cisgender women, Transgender men		

					agency manager who was also an online sex worker)			
Beksinska, 2021	Prevalence and Correlates of Common Mental Health Problems and Recent Suicidal Thoughts and Behaviours among Female Sex Workers in Nairobi, Kenya	African Region, Kenya	Cross-sectional, Observational	1003; simple random sampling	Not defined	Cisgender women	Depression 23%; Anxiety 11%; PTSD 14%; Suicidal ideation and/or suicide attempts 10%	
MacLean, 2017	Prevalence and Correlates of Probable Depression and Post-Traumatic Stress Disorder among Female Sex Workers in Lilongwe, Malawi	African Region, Malawi	Cross-sectional, Observational	200; venue based sampling	Defined as someone "who had received money in exchange for sex either regularly or occasionally up to 12 months"	Cisgender women	Depression 8%; PTSD 8%; Suicidal ideation 3%	
Ouma, 2021	Prevalence and Factors Associated with Major Depression among Female Sex Workers in Post-Conflict Gulu District: A Cross-Sectional Study	African Region, Uganda	Cross-sectional, Observational	300; simple random sampling	Not defined	Cisgender women	Depression 48%	Fertility intentions† 55%
Roshanfekr, 2023	Prevalence and predictor factor of lifetime abortion in female sex workers in Iran: results of the national rapid assessment and response in 2017	Eastern Mediterranean Region, Iran	Cross-sectional, Observational	855; N/A	Defined as had commercial sex within 12 months prior to the interview	Cisgender women		Termination 41%
Long, 2019	Prevalence and predictors of unmet contraceptive need in HIV-positive female sex workers in Mombasa, Kenya	African Region, Kenya	Cross-sectional, Observational	346; Venue-based sampling	Defined as women who reported exchanging sex for cash or in-kind payment at the time of enrollment.	Cisgender women	Depression 32%	Unmet need for contraception 64%; Fertility intentions† 19%
Bhardwaj, 2023	Prevalence of Depression, Syndemic Factors and their Impact on Viral Suppression among Female Sex Workers Living with HIV in Ethekwini, South Africa	African Region, South Africa	Cross-sectional, Observational	1384; venue based sampling; convenience sampling	Not defined	Cisgender women	Depression 33%	
Pahwa, 2024	Prevalence of human papilloma virus infection and risk of cervical intraepithelial neoplasia among female sex workers in Mumbai, India	South-East Asia Region, India	Cross-sectional, Observational	448; Convenience Sampling	Not defined	Cisgender women		Termination 40%; HPV infection 36%
Iaisuklang, 2017	Psychiatric Morbidity among Female Commercial Sex Workers	South-East Asia Region, India	Cross-sectional, Observational	100; simple random sampling	Not defined	Cisgender women	Depression 9%; Anxiety 8%; PTSD 21%	
Patel, 2016	Relationship Between Mobility, Violence and Major Depression among Female Sex Workers: A Cross-Sectional Study in Southern India	South-East Asia Region, India	Cross-sectional, Observational	2400; two-stage cluster random sampling	Defined as those who are registered as a sex worker by a CBO	Cisgender women	Depression 29%	

Yam, 2019	Reproductive health and desire for children among young female sex workers in Bangladesh brothels	South-East Asia Region, Bangladesh	Cross-sectional, Quasi-experimental	1061; Venue-based Sampling	Defined as female residents in the study brothels who exchanged sex for money in the past 30 days	Cisgender women, Transgender men		Fertility intentions† 55%; Termination 12%
Rogers, 2015	Risk indicators of Depressed Mood among Sex-Trade Workers and Implications For HIV Risk Behaviour	Region of the Americas, Canada	Cross-sectional, Observational	299; venue based sampling	Defined as those who answered yes to the question, “Have you ever traded sex for money?”	Cisgender women, Cisgender men, Transgender women, Transgender men, Nonbinary/Other	Depression 85%	Fertility intentions† 40%
Yam, 2020	Safer conception for female sex workers living with HIV in Dar es Salaam, Tanzania: Cross-sectional analysis of needs and opportunities in integrated family planning/HIV services	African Region, Tanzania	Cross-sectional, Observational	300; Convenience Sampling	Defined as women who reported that they receive money, goods, or favors in exchange for sexual services as a primary source of income [i.e., more than half their monthly income	Cisgender women		Fertility intentions† 29%
Krumrei-Mancuso , 2016	Sex Work and Mental Health: A Study of Women in the Netherlands	European Region, Netherlands	Cross-sectional, Observational	88; venue based sampling and convenience sampling	Not defined	Cisgender women		
Duff, 2015	Sex work and motherhood: social and structural barriers to health and social services for pregnant and parenting street and off-street sex workers	Region of the Americas, Canada	Cross-sectional, Observational	399; Time-location Sampling	Not defined	Cisgender women, Transgender women		
Drydakis, 2021	Sex Workers’ Self-Reported Physical and Mental Health in Greece. A Repeated Cross-Sectional Study in 2009, 2013 and 2019	European Region, Greece	Cross-sectional, Observational	352; venue based sampling	Defined as a legal sex worker who is registered with the local prefecture, carries a valid medical card, and works in a licensed establishment	Cisgender women, Cisgender men, Transgender women, Transgender men		
Slabbert, 2017	Sexual and reproductive health outcomes among female sex workers in Johannesburg and Pretoria, South Africa: Recommendations for public health programmes	African Region, South Africa	Cross-sectional, Observational	1830; Venue-based Sampling	Defined as women engaged as commercial sex workers	Cisgender women		
LaFort, 2017	Sexual and reproductive health services utilization by female sex workers is context-specific: results from a cross-sectional survey in India, Kenya, Mozambique and South Africa	African Region, South-East Asia Region, India; Kenya; Mozambique; South Africa	Cross-sectional, Observational	1566; respondent-driven sampling	Defined as females 18 years or older who had received money or gifts for sex at least three times in the last 6 months.	Cisgender women, Transgender men		Termination 15%-94%; Cervical cancer screening 29%

Becquet, 2020	Sexual health needs of female sex workers in Côte d'Ivoire: a mixed-methods study to prepare the future implementation of pre-exposure prophylaxis (PrEP) for HIV prevention	African Region, Cote D'Ivoire	Cross-sectional, Observational	1000; Convenience Sampling	Not defined	Cisgender women		Termination 50%
Braga, 2021	Sexual, reproductive health and health status of female sex workers in 12 Brazilian cities, 2016	Region of the Americas, Brazil	Cross-sectional, Observational	4328; Respondent-driven Sampling	Defined as women aged 18 years old or older, who worked as sex worker who had at least one sexual intercourse in exchange for money in the four months prior to the survey	Cisgender women	Depression 30%	Antenatal and/or postnatal care 61%-99%; Cervical cancer screening 53%-73%
Kilembe, 2019	Single mothers and female sex workers in Zambia have similar risk profiles	African Region, Zambia	Cross-sectional, Observational	731 in Lusaka; 646 in Ndola; Clinical Referral; Venue-based Sampling	Not defined	Cisgender women		
Zehnder, 2019	Stigma As A Barrier To Mental Health Service Use among Female Sex Workers in Switzerland	European Region, Switzerland	Cross-sectional, Observational	60; Convenience sampling	Not defined	Cisgender women	Depression 54%	
Goldenberg, 2023	Stigma, Social Cohesion, and Mental Health among Transgender Women Sex Workers Living with HIV in the Dominican Republic	Region of the Americas, Dominican Republic	Cross-sectional, Observational	100; convenience sampling	Defined as those who are reporting exchanging sex for money in the past month	Transgender women	Depression 24%; Anxiety 34%	
Wang, 2022	Substance Use and Depression Impede Art Adherence among Female Sex Workers Living with HIV in the Dominican Republic	Region of the Americas, Dominican Republic	Longitudinal, Observational	240; Convenience sampling	Defined as cisgender women who reported exchanging sex for money in the past month.	Cisgender women	Depression 70%	
Macias-Konstantopoulos, 2024	Suicide During Pregnancy as a Major Contributor to Maternal Suicide among Female Sex Workers in Eight Low- and Middle-income Countries: A Community Knowledge Approach Investigation	African Region; Region of the Americas; South-East Asia Region, Angola; Brazil; the Democratic Republic of the Congo; India; Indonesia; Kenya; Nigeria; South Africa	Cross-sectional, Observational	1280 FSW reporting on 2112 FSW deaths; snowball sampling	Defined as those who have practiced full-time sex work for at least 3 years	Cisgender women	Suicide deaths 14%	
Chandler, 2020	Syndemic Health Disparities and Sexually Transmitted infection Burden among Black Men who Have Sex with Men Engaged in Sex Work in the U.S.	Region of the Americas, United States	Cross-sectional, Observational	254; time location sampling	Defined as those who report receiving money, drugs, or other goods for sex with either male or female clients	Cisgender men, Transgender women	Depression 59%	
Couture, 2020	Syndemic Psychosocial Health Conditions Associated with Recent Client-Perpetrated Violence Against Female Entertainment and Sex Workers in Cambodia	Western Pacific Region, Cambodia	Cross-sectional, Cluster Randomized Stepped-Wedge Trial	1198; convenience sample	Defined as those who have sex in exchange for money, goods, services or drugs within the past month	Cisgender women		

Carlson, 2017	The Impact of Violence, Perceived Stigma, and Other Work-Related Stressors on Depressive Symptoms among Women Engaged in Sex Work	Western Pacific Region, Mongolia	Cross-sectional, Observational	222; purposive sampling	Defined as women at least 18 years of age; reported having engaged in vaginal or anal sexual intercourse in the 90 days prior to screening in exchange for money, alcohol, or other goods; or with a paying sexual partner;	Cisgender women	Depression 60%	
Liang, 2024	The prevalence and correlates of unintended pregnancy among female sex workers in South China: a cross-sectional study	Western Pacific Region, China	Cross-sectional, Observational	1257; Purposive sampling	Defined as females assigned at birth, 18 years of age or older, who had traded sex for money or goods in the past year	Cisgender women, Transgender men		
Duff, 2015	The relationship between social, policy and physical venue features and social cohesion on condom use for pregnancy prevention among sex workers: a safer indoor work environment scale	Region of the Americas, Canada	Longitudinal, Observational	588; Time-Location Sampling	Defined as cisgender and transgender women that exchanged sex for money within the last 30 days in street, indoor and online venues	Cisgender women, Transgender women		
Grosso, 2023	the Relationship Between Underage initiation of Selling Sex and Depression among Female Sex Workers in Eswatini	African Region, Eswatini	Cross-sectional, Observational	767; snowball sampling	Defined as those who have exchanged sex for money, favors, or goods in the past 12 months	Cisgender women	Depression 43%	
Tounkara, 2021	Type-specific incidence, persistence and factors associated with human papillomavirus infection among female sex workers in Benin and Mali, West Africa	African Region, Benin; Mali	Longitudinal, Observational	665 FSW: 209 with no follow up visits, 456 with at least one follow up visit; Venue-based Sampling	Not defined	Cisgender women		HPV infection 87%
Katz, 2015	Understanding the broader sexual and reproductive health needs of female sex workers in Dhaka, Bangladesh	South-East Asia Region, Bangladesh	Cross-sectional, Observational	354 hotel based sex workers, 323 street based sex workers; Venue-based Sampling	Defined as any woman who reported having received money or goods in exchange for sexual ser- vices as a source of income in the last six months	Cisgender women		Unmet need for contraception 36%; Termination 41%
Twizelimana, 2021	Unmet contraceptive needs among female sex workers (FSWs) in semi urban Blantyre, Malawi	African Region, Malawi	Cross-sectional, Observational	290; Systematic sampling	Defined as “self-reported"	Cisgender women		Unmet need for contraception 35%
Bowring, 2020	Unmet need for family planning and experience of unintended pregnancy among female sex workers (FSWs) in urban Cameroon: results from a national cross-sectional study	African Region, Cameroon	Cross-sectional, Observational	2255; Respondent-driven Sampling	Not defined	Cisgender women		Fertility intentions† 64%; Termination 43%
Akolo, 2024	Uptake of cervical cancer screening among sex workers living with HIV in Nairobi, Kenya: a cross-sectional study	African Region, Kenya	Cross-sectional, Observational	75; Simple random sampling	Defined as attending SWOP clinic	Cisgender women		Cervical cancer screening 45%
Santos-Couto, 2019	Use of hormonal contraceptives by prostitutes: correlation with markers of social vulnerability	Region of the Americas, Brazil	Cross-sectional, Observational	69; Convenience Sampling	Defined as in professional practice	Cisgender women		

Namale, 2021	Visual inspection with acetic acid (VIA) positivity among female sex workers: a cross-sectional study highlighting one-year experiences in early detection of pre-cancerous and cancerous cervical lesions in Kampala, Uganda	African Region, Uganda	Cross-sectional, Observational	719; Convenience Sampling	Defined as women having sex with men in exchange for money, favours or other goods either regularly or casually at least once in the past 12 months.	Cisgender women		

*Studies reporting proportions for mental health outcomes are presented in S2 Table. Data are not presented for studies that reported mental health outcomes continuously (e.g., scores) in the absence of a proportion.

†Fertility intentions have been aggregated to indicate those participants that expressed plans to become pregnant, either currently or at some point in the future.
